# Efficient Visible Light Photocatalytic Hydrogen Evolution by Boosting the Interfacial Electron Transfer in Mesoporous Mott–Schottky Heterojunctions of Co_2_P-Modified CdIn_2_S_4_ Nanocrystals

**DOI:** 10.1021/acsaem.4c00710

**Published:** 2024-05-21

**Authors:** Evangelos K. Andreou, Ioannis Vamvasakis, Gerasimos S. Armatas

**Affiliations:** Department of Materials Science and Engineering University of Crete, Vassilika Vouton, Heraklion 70013, Greece

**Keywords:** thiospinels, cobalt phosphide, nanoporous materials, photocatalysis, hydrogen evolution

## Abstract

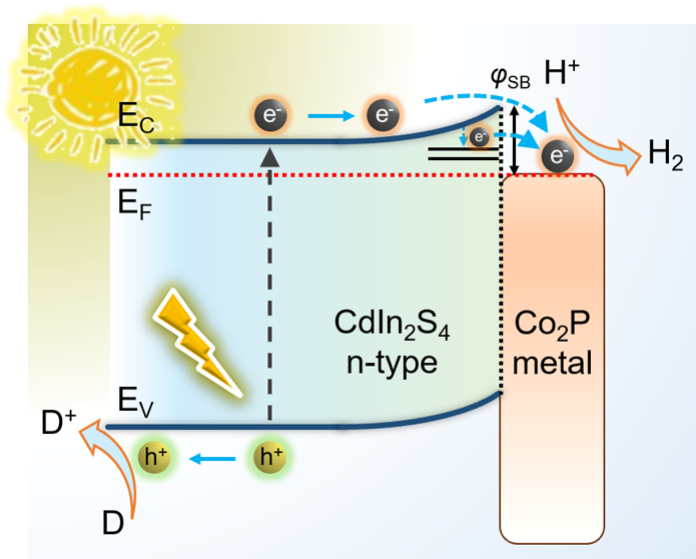

Photocatalytic water splitting for hydrogen generation is an appealing means of sustainable solar energy storage. In the past few years, mesoporous semiconductors have been at the forefront of investigations in low-cost chemical fuel production and energy conversion technologies. Mesoporosity combined with the tunable electronic properties of semiconducting nanocrystals offers the desired large accessible surface and electronic connectivity throughout the framework, thus enhancing photocatalytic activity. In this work, we present the construction of rationally designed 3D mesoporous networks of Co_2_P-modified CdIn_2_S_4_ nanoscale crystals (ca. 5–6 nm in size) through an effective soft-templating synthetic route and demonstrate their impressive performance for visible-light-irradiated catalytic hydrogen production. Spectroscopic characterizations combined with electrochemical studies unravel the multipathway electron transfer dynamics across the interface of Co_2_P/CdIn_2_S_4_ Mott–Schottky nanoheterojunctions and shed light on their impact on the photocatalytic hydrogen evolution chemistry. The strong Mott–Schottky interaction occurring at the heterointerface can regulate the charge transport toward greatly improved hydrogen evolution performance. The hybrid catalyst with 10 wt % Co_2_P content unveils a H_2_ evolution rate of 20.9 mmol g_cat_^–1^ h^–1^ under visible light irradiation with an apparent quantum efficiency (AQE) up to 56.1% at 420 nm, which is among the highest reported activities. The understanding of interfacial charge-transfer mechanism could provide valuable insights into the rational development of highly efficient catalysts for clean energy applications.

## 1 Introduction

The current dependence on fossil fuels to fulfill the growing energy requirements is a major burden on the economy and environment, which triggers keen research for alternative cost-effective and cleaner energy sources.^1^ Inspired from photosynthesis in nature, photocatalysis is an appealing method to generate renewable fuels by direct utilization of solar energy.^2,3^ Hydrogen (H_2_) is an excellent energy carrier due to its high gravimetric energy density (120 MJ kg^–1^) and zero-carbon emission when converted to electricity or heat. Since the water splitting is an uphill reaction, a lot of effort has been made over the past years in developing robust and highly active semiconductor catalysts for promoting the hydrogen production reaction.^4^ Thus far, a large number of transition metal oxides (Nb_2_O_5_, SrTiO_3_, Cu_2_O, WO_3_, etc.),^5−7^ chalcogenides (CdS, Bi_2_S_3_, ZnS, MoS_2_, etc.),^8−10^ and (oxy)nitrides (TaON, BaTaO_2_N, LaTiO_2_N, etc.)^11,12^ with desired structures and electronic properties have been synthesized. Nevertheless, the majority of these materials have achieved ambiguous success in hydrogen generation applications largely due to their rapid electron–hole recombination, poor photochemical stability, and high cost.^13^ Metal sulfides have gained tremendous interest in the field of photocatalytic hydrogen production among various photocatalysts owing to their outstanding properties, including tunable band gap, wide light absorption, multiple redox activity, high photoinduced electron transfer (typically > 20 cm^2^ V^–1^ s^–1^), and well-suited band structure for water splitting. Moreover, coupling the metal chalcogenide structure with another metal or semiconductor to form a heterostructure with suitable band-edge positions has been proven as an effective strategy to enhance the charge carrier separation efficiency.

Thiospinel compounds, such as ZnIn_2_S_4_, NiFe_2_S_4_, and CuCo_2_S_4_, have recently become heavily studied materials for energy harvesting and conversion applications, bringing intriguing perspectives to the fields of water electrolysis, electrochemical reduction of CO_2_, and polymer electrolyte fuel cells.^14−16^ Many of these materials benefit from a high visible light absorption coefficient (typically ca. 10^4^–10^5^ cm^–1^), high electrical conductivity as compared to transitional metal oxides and other chalcogenides, and great photostability, especially in harsh electrolytes.^17,18^ Though promising, the actual photocatalytic performance of thiospinel materials is often limited by the severe charge recombination, low density of active sites, and ensuing insufficient utilization of the surface-reaching carriers, which are crucial to optimizing the charge transport and reaction kinetics. The rational design of catalysts with a nanoscale morphology and large surface area is a highly competitive strategy for advancing photocatalytic efficiency. However, the synthesis of thiospinel nanostructures with an open-pore architecture is negatively affected by the low chemical affinity of transition metals to sulfur and large lattice contraction during the crystallization process, which inevitably result in structural collapse of the thiospinel products with limited porosity (low internal surface area).

Recently, we devised a facile synthetic route to assemble mesoporous structures of hexagonal CdIn_2_S_4_ nanocrystals (NCs) using colloidal chemistry and polymer-assisted self-assembly methods.^19^ Unlike the conventional high-temperature solid-state and solvothermal methods,^20−22^ this synthetic approach enables us to fabricate nanostructured materials with large internal surface area, controllable grain composition, and high photocatalytic reactivity. Herein, we couple polymer-templating self-assembly synthesis and a wet-chemical deposition method to produce new high-surface-area mesoporous semiconducting CdIn_2_S_4_ frameworks decorated with highly dispersed metallic Co_2_P nanoparticles for efficient photocatalytic hydrogen evolution. That involves the chemical deposition of Co_2_P on assembled CdIn_2_S_4_ mesostructures. To date, constructing Co_2_P/CdIn_2_S_4_ Mott–Schottky heterojunctions for efficient photocatalytic hydrogen production has not yet been reported. The hybridization with metal phosphide nanocrystals enables the heterostructures to achieve improved photocatalytic efficiency through the charge carrier dissociation and transportation. Metal-rich phosphides, such as Co_2_P, Cu_3_P, and Ni_2_P, have recently emerged as highly active electro- and photocatalysts because of their high intrinsic carrier mobility and great chemical stability (both in acidic and alkaline solutions).^23−26^ We show that these Co_2_P/CdIn_2_S_4_ Mott–Schottky junctions can exhibit excellent electron transfer along the porous framework to the surface-dispersed Co_2_P nanoparticles that remarkably reduces interfacial charge recombination at the catalyst/electrolyte contact, which is essential for boosting the reaction kinetics. Consequently, modification of CIS mesostructure with 10% by weight (wt %) Co_2_P brings an encouraging hydrogen evolution rate of 20.9 mmol g_cat_^–1^ h^–1^ associated with an apparent quantum efficiency of 56.1% at 420 nm, which is among the best activities reported for thiospinel-based catalysts. By combining photoluminescence with (photo)electrochemical spectroscopy measurements, we unveil a pertinent mechanistic scheme for the charge transfer dynamics and hydrogen evolution reaction in these heteronanostructures.

## 2 Results and Discussion

### 2.1 Synthesis and Structural Characterization

Mesoporous frameworks of linked CdIn_2_S_4_ nanocrystals (denoted as CIS NCFs) were synthesized through the oxidative coupling of colloidal CIS nanoparticles in the presence of a polyoxoethylene-*b*-cetyl ether (Brij-58) block copolymer template, which is sufficient to mediate the assembly of individual nanoparticles into extended 3D mesostructures.^27,28^ The polymer template was then carefully eliminated from the framework by solvent extraction in warm ethanol and water (∼40 °C) to recover the mesoporous CIS product. This process produces a robust network of connected CIS nanocrystals with a large internal surface area and well-defined pores. The extraction of the organic components was verified by thermogravimetric analysis (TGA). The TGA plot of the solvent-treated CIS sample indicated an inevitable ∼9.3 wt % of organic residue remaining in the pores (Figure S1, Supporting Information). Then, mesoporous Co_2_P-modified CIS heterostructures (denoted as *n*-CP/CIS NCFs) with adjustable composition of Co_2_P (*n*), that is, from 5 to 15 wt %, were prepared by chemical deposition of preformed Co_2_P nanoparticles on the surface of CIS NCFs. As a control sample, CIS microparticles were also prepared through a well-established hydrothermal synthesis, followed by chemical deposition of 10 wt % Co_2_P to form Co_2_P/CIS bulk material (denoted as 10-CP/CIS_*m*, see the Section 4). Energy-dispersive X-ray spectroscopy (EDS) measurements confirmed that the resultant CP/CIS NCFs have an elemental composition of Cd:In:S close to 1:2:4 and Co_2_P loadings very similar to those expected from the stoichiometry of reactions (within a 5–8% standard deviation, see Figure S2, Supporting Information). The analytical data and the estimated Co_2_P loadings of mesoporous samples are given in Supporting Table S1.

**Figure 1:**
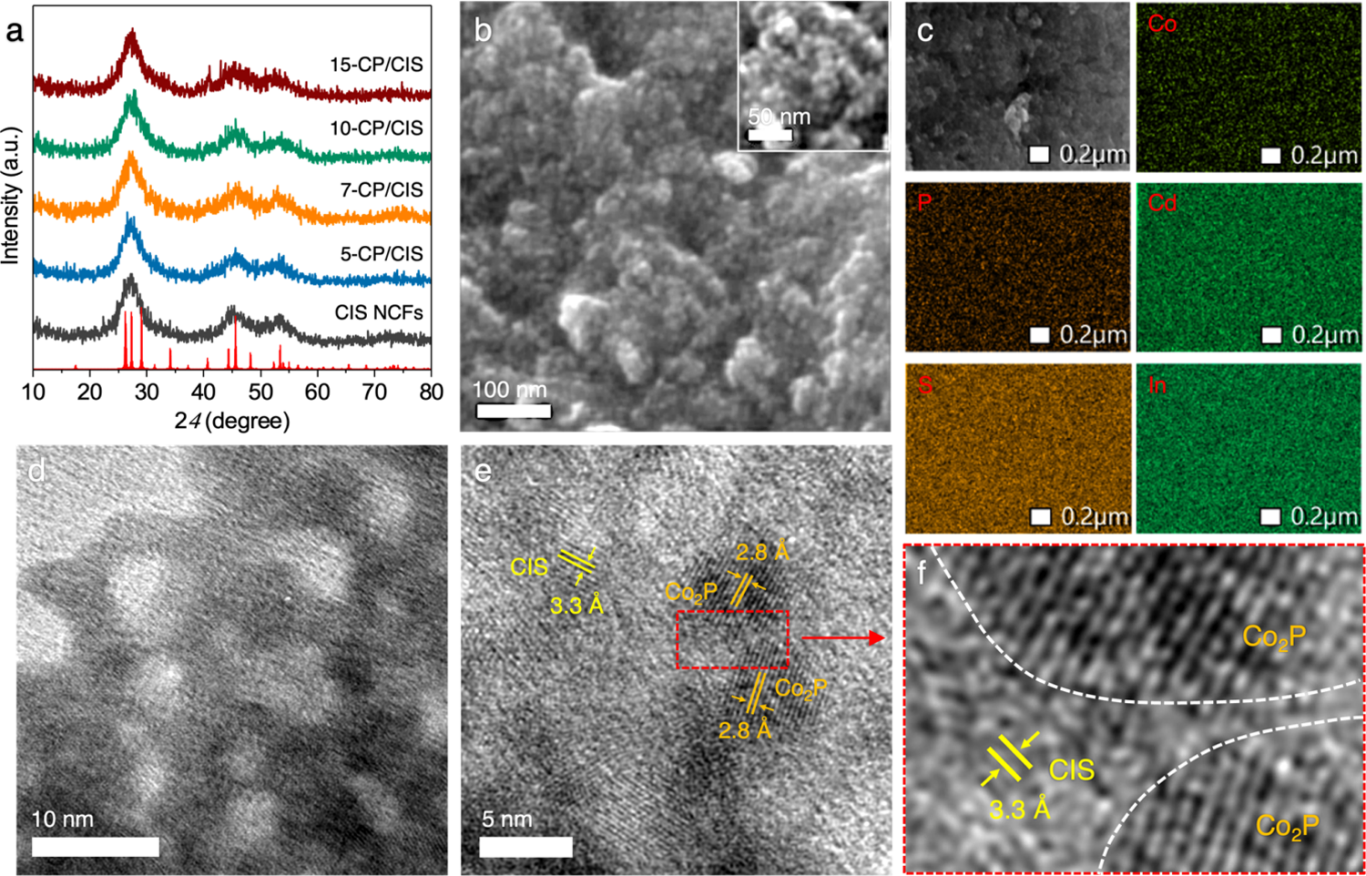
(a) XRD patterns of mesoporous CIS and CP/CIS NCFs. (b) FESEM image (Inset: magnified image), (c) EDS elemental mappings of Cd, In, S, Co, and P elements, and (d) TEM and (e, f) high-resolution TEM (HRTEM) images of the mesoporous 10-CP/CIS NCFs. In panel (a), the standard diffraction lines of hexagonal CdIn_2_S_4_ (space group *P*6_3_*mc*; red lines) are also given.

The crystal structures of mesoporous CIS and CP/CIS NCFs were verified via X-ray diffraction (XRD) analysis. The CIS NCFs exhibited three broad diffraction peaks at 2θ scattering angles from 20 to 60° (Figure 1a), which, according to previous investigations, belong to the hexagonal phase of CdIn_2_S_4_.^19^ The presence of broad diffraction peaks suggests that the materials are nanocrystalline in nature. The mesoporous CP/CIS NCFs with Co_2_P content up to 10 wt % displayed a similar XRD pattern with the pristine CIS with no detectable Co_2_P crystallites due to the small grain size and low content of Co_2_P. The high Co_2_P-loaded sample (15 wt %), however, apart from the XRD peaks belonging to CIS, showed an additional diffraction peak at 2θ ∼ 40.7°, which can be indexed to the (112) lattice planes of orthorhombic Co_2_P, confirming the formation of the Co_2_P/CIS heterostructure. The crystal phase of as-made Co_2_P was identified by XRD as shown in Supporting Figure S3, where all of the diffraction peaks are consistent with the orthorhombic phase of Co_2_P (JCPDS card no. 32–0306). Besides, no other distinguishable XRD peaks were detected, pointing to the phase purity of Co_2_P. The XRD pattern of bulk CIS synthesized by the hydrothermal method was recognized as the standard cubic CdIn_2_S_4_ structure (JCPDS card no. 27–0060). (Figure S4, Supporting Information).

Field emission scanning electron microscopy (FESEM) characterization of the mesoporous sample with 10 wt % Co_2_P content (10-CP/CIS NCFs), which is the most active catalyst toward hydrogen evolution, discloses the formation of a 3D porous network consisting of fairly monodisperse nanoparticles within the range of 5–10 nm (Figure 1b). FESEM combined with EDS spectroscopy of the 10-CP/CIS NCFs is also reported in Figure 1c, revealing a homogeneous distribution of Cd, In, Co, S, and P elements throughout the composite framework. These elemental mapping images demonstrate that Co_2_P deposits uniformly on the CIS host structure, which can amplify exposure of available active sites. Further structural characterization of 10-CP/CIS NCFs with transmission electron microscopy (TEM) displays plenty of tightly interconnected nanoparticles of 5–7 nm diameter forming the porous framework (Figure 1d), in accordance with the results from XRD and FESEM. Such an intimate particle-to-particle contact is crucial to maximize the electrical conductivity of the mesoporous structure. Moreover, the TEM image in Figure 1d also displays uniform pores of about 4–5 nm in size embedded within the assembled structure, which is beneficial to the mass transfer process. Further evidence that the constituent nanocrystals do adopt a hexagonal crystal structure is shown in the high-resolution TEM (HRTEM) image. The distinct lattice fringes with an interplanar distance of 3.3 Å in Figure 1e are assigned to the (011) crystal planes of hexagonal CdIn_2_S_4_ (space group *P*6_3_*mc*).^19^ The different orientation of the lattice planes suggests the polycrystalline nature of CIS. Furthermore, HRTEM investigation also verifies that Co_2_P nanoparticles are well dispersed on the CIS surface, clearly showing Co_2_P nanocrystals (appeared as dark regions) with lattice plane distance of 2.8 Å, which agree well with the (200) crystal planes of the orthorhombic Co_2_P (space group *Pnma*). Careful analysis of the TEM images suggests that Co_2_P comprises nanocrystallites with an average grain size of ca. 6–7 nm. The HRTEM also showed distinct heterointerfaces between the Co_2_P and CIS phases (marked with white lines in Figure 1f), which are anticipated to be beneficial for the dissociative adsorption of water molecules during the hydrogen evolution reaction.^29^ Taken together with EDS and XRD characterizations of chemical composition and crystallinity, these results are entirely consistent with the fabrication of porous frameworks comprising interconnected Co_2_P and CIS nanocrystals with internal porosity, which allows for an efficient interparticle contact and maximizes the electrical conductivity.

**Figure 2:**
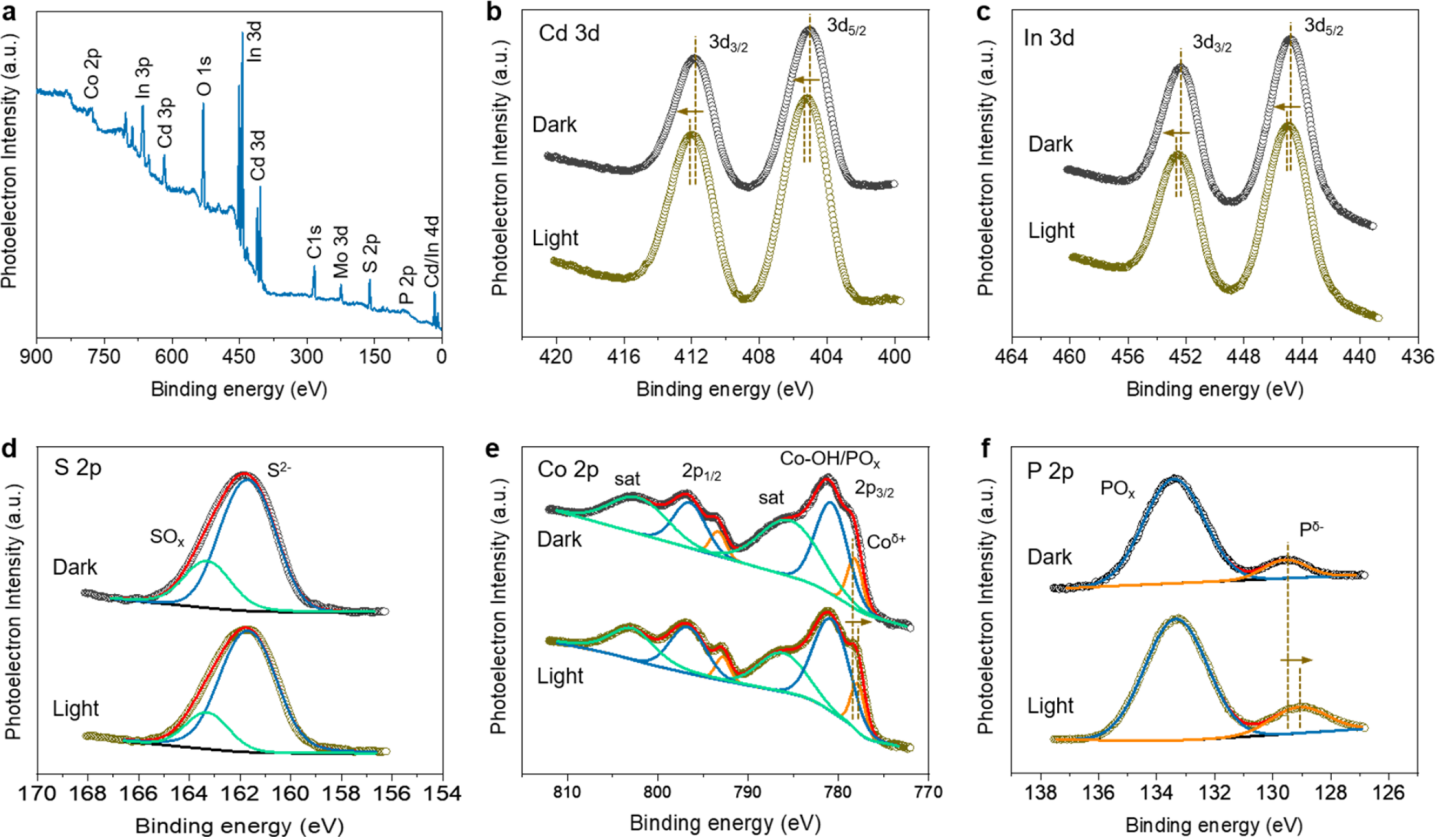
(a) XPS survey scan and high-resolution XPS spectra of (b) Cd 3d, (c) In 3d, (d) S 2p, (e) Co 2p, and (f) P 2p core levels of mesoporous 10-CP/CIS NCFs. In situ XPS spectra were collected under visible light irradiation. The signal of Mo 3d in panel (a) is from the sample holder. The XPS deconvoluted spectra of different chemical species are shown as blue, green, and orange curves. The red lines are fits to the experimental data.

X-ray photoelectron spectroscopy (XPS) further confirmed the successful construction of the Co_2_P-CdIn_2_S_4_ heterostructures. The XPS survey spectrum obtained from the 10-CP/CIS NCFs sample verifies the presence of Cd, In, S, O, Co, and P elements (Figure 2a), in line with the results of EDS analysis. Note that the O signal could be due to partial oxidation of surface atoms as a result of sample exposure to air. The Cd 3d XPS spectrum of 10-CP/CIS NCFs showed a doublet peak for the Cd 3d_5/2_ and Cd 3d_3/2_ spin–orbit states at 405.0 and 411.8 eV, respectively (Figure 2b), consistent with the divalent Cd in metal sulfides, suggesting the existence of Cd–S bonds.^30,31^ Similarly, in the In 3*d* region, the pair of peaks located at 444.8 and 452.4 eV are identified as In^3+^ 3d_5/2_ and In^3+^ 3d_3/2_ spin–orbit lines, respectively, confirming the +3 valence state of In (Figure 2c). The S 2p deconvoluted peaks at 161.7 and 163.3 eV binding energies are consistent with the literature values for S–metal and S–O(OH) bonds, respectively (Figure 2d).^32^ The low-valence states of Co^δ+^ and P^δ−^ in Co_2_P can be affirmed by the Co 2p_3/2_ and P 2p core-level signals at 778.2 and 129.6 eV, respectively. Besides, other Co 2p_3/2_ and P 2p subpeaks at 780.8 and 133.4 eV were discerned, being ascribed to the Co–OH/Co–PO_*x*_ bonds. These binding energies imply the surface of Co_2_P is partially oxidized likely due to exposure to air (Figure 2e,f).^33−35^ The EDS evidence for the nominal composition of 10-CP/CIS NCFs is in agreement with the quantitative analysis from XPS, which indicates a surface Co/In ratio of ∼0.28 that corresponds to an ∼8.3 wt % Co_2_P loading amount. We also measured in situ irradiated XPS (ISI-XPS) to understand the charge transfer interactions between the CIS and Co_2_P under light irradiation. As revealed in Figure 2b,c,e,f, during the photoexcitation of 10-CP/CIS NCFs, a positive shift (by ∼0.2 eV) of the Cd 3d_5/2_ (405.2 eV) and In 3d_5/2_ (445.0 eV) XPS lines have been observed, along with a concurrent negative shift (by ca. 0.3–0.5 eV) of the Co 2p_3/2_ (777.9 eV) and P 2p_3/2_ (129.1 eV) XPS lines, in comparison to those in the corresponding ex situ (in dark conditions) spectra. These binding energy shifts unequivocally confirm a photoelectron transition from Cd/In 3d states of CIS to Co/P 2p states of Co_2_P through the interface. Such a charge redistribution is favorable for the spatial separation of excitons, improving the photocatalytic efficiency.

**Figure 3:**
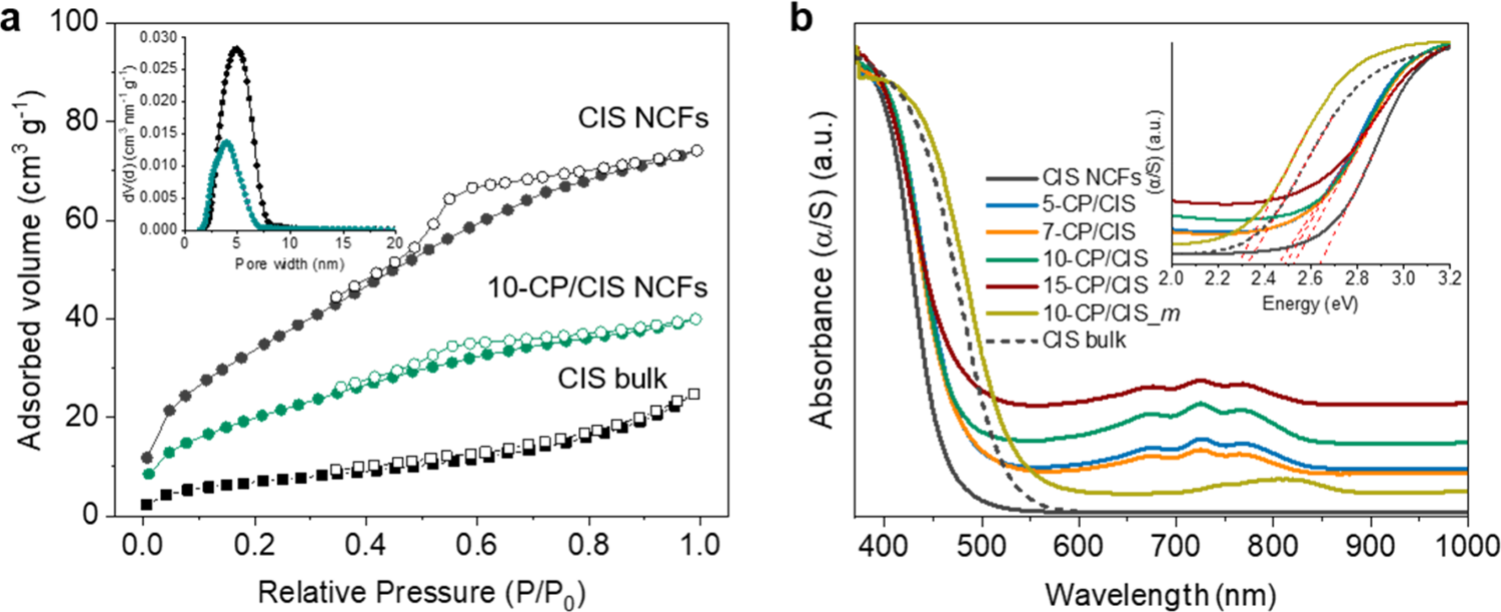
(a) N_2_ adsorption (filled cycles) and desorption (open cycles) isotherms at −196 °C for the mesoporous CIS and 10-CP/CIS NCFs and bulk CIS. The inset shows the corresponding NLDFT pore size distributions calculated from the adsorption isotherms. (b) UV–vis/NIR diffuse reflectance spectra indicating the effect of different Co_2_P loadings on the optical absorption of the CIS mesoporous structure. The UV–vis/NIR absorption spectra of the CIS and 10-CP/CIS_*m* bulk reference samples are also given. The inset shows the corresponding Kubelka–Munk versus energy plots.

The permanent porosity of the as-prepared samples was probed with nitrogen physisorption measurements. The N_2_ adsorption–desorption isotherms of the mesoporous CIS are compared in Figure 3a to 10-CP/CIS NCFs and bulk CIS, while the N_2_ isotherms of the other CP/CIS heterostructures are depicted in Supporting Figure S5. All of the isotherms exhibit a typical type-IV shape associated with an H_2_-type hysteresis loop according to IUPAC classification, indicating mesoporous structures with interconnected pores.^36^ The distinct hysteresis loop between the adsorption and desorption branches implies the presence of pore blocking effects produced by cage-type or split-shaped pores. The CIS NCFs display a Brunauer–Emmett–Teller (BET) surface area of 132 m^2^ g^–1^ and a pore volume of 0.12 cm^3^ g^–1^, which are very large if we account for the heavier inorganic framework. After decoration with Co_2_P, the BET surface area (55–106 m^2^ g^–1^) and pore volume (0.05–0.08 cm^3^ g^–1^) of the CP/CIS composites gradually decrease with increasing Co_2_P content due to the heavy atoms of Co_2_P nanocrystals that are introduced onto the framework. The analysis of the adsorption data with the non-local density functional theory (NLDFT) method gives quite narrow size distributions of pores with a pore diameter at ∼5 nm for CIS NCFs and ca. 3.8–4.0 nm for CP/CIS NCFs, in accordance with the interstitial pore spaces observed by TEM (ca. 4–5 nm). The gradual decrease in surface area and pore size for Co_2_P-modified samples is consistent with the deposition of Co_2_P nanoparticles inside the pores of the CIS NCFs. Even so, all of the CP/CIS NCFs still consistently display adequate open-pore structure with a highly accessible surface area. Noticeably, the surface areas of bulk CIS and 10-CP/CIS_*m* microparticles prepared by hydrothermal synthesis are very low (ca. 25 and 9 m^2^ g^–1^, respectively, see Figure S5d, Supporting Information), indicative of nonporous morphologies. All textural parameters of the CP/CIS NCFs along with those of pristine CIS NCFs and bulk CIS and 10-CP/CIS_*m* materials are given in Table 1.

**Table 1:** Textural Properties and Optical Band Gaps of the Mesoporous CIS and CP/CIS NCFs, and CIS and 10 wt % Co_2_P-Loaded CIS Microparticles

catalyst	BET surface area (m^2^ g^–1^)	pore volume (cm^3^ g^–1^)	pore size (nm)	energy gap (eV)
CIS NCFs	132	0.12	5.0	2.63
5-CP/CIS NCFs	106	0.08	4.0	2.51
7-CP/CIS NCFs	87	0.07	3.9	2.49
10-CP/CIS NCFs	75	0.06	4.0	2.50
15-CP/CIS NCFs	55	0.05	3.8	2.42
CIS bulk	25	0.03		2.30
10-CP/CIS_*m*	9	<0.01		2.26

The optical properties of the mesoporous CIS and CP/CIS NCFs were characterized by ultraviolet–visible/near-IR (UV–vis/NIR) diffuse reflectance spectroscopy. The CIS NCFs showed a sharp optical absorption onset at ∼471 nm (2.63 eV), which is attributed to the band-edge transition in CdIn_2_S_4_ (Figure 3b). The small size of the constituent CIS nanoparticles (ca. 5–7 nm according to TEM observation) has a prompt effect on the band gap energy (*E*_g_), giving rise to larger *E*_g_ values than that of bulk CIS (2.32 eV). Generally, such a blue shift in the optical absorption spectra is a fascinating feature of nanomaterials and it can be elucidated by size-induced quantum confinement effects, analogous to those realized in discrete nanoparticles and nanosized frameworks.^37^ In comparison with pristine CIS NCFs, the Co_2_P decorated samples showed a slightly narrower energy gap by ca. 100–200 meV; the *E*_g_ of CP/CIS NCFs varies systematically with the Co_2_P composition from 2.51 eV for the 5 wt % Co_2_P to 2.42 eV for the 15 wt % Co_2_P-modified sample. This variation of the energy gap (red shift) arises from the strong interfacial electronic interaction between Co_2_P and the CIS host material after hybridization. Besides, the incorporation of Co_2_P onto the CIS structure leads to an increase in the visible/near-IR light absorption owing to the light-harvesting capability (d–d charge-transfer transitions of tetrahedral Co clusters) of Co_2_P.^38^ Notably, the as-synthesized Co_2_P shows a continuous absorption over the entire UV–visible/NIR wavelength range (Figure S6, Supporting Information), which supports metallic behavior. The metallic character of Co_2_P was also verified by electrochemical measurements (see below). It is worth noting that the *E*_g_ of 10-CP/CIS_*m* is about 300 meV smaller (2.26 eV) than the energy band gap of mesoporous 10-CP/CIS NCFs, reasonably due to the larger grain size composition. FESEM images in Supporting Figure S7a show that the hydrothermally obtained CIS sample has a microstructural morphology with particle size ranging between 8 and 10 μm. The magnified FESEM image in Supporting Figure S7b also discloses that the individual CIS microparticles are composed of plenty of intersecting ∼250 nm-sized nanoflakes.

### 2.2 Photocatalytic Hydrogen Evolution Study

**Figure 4:**
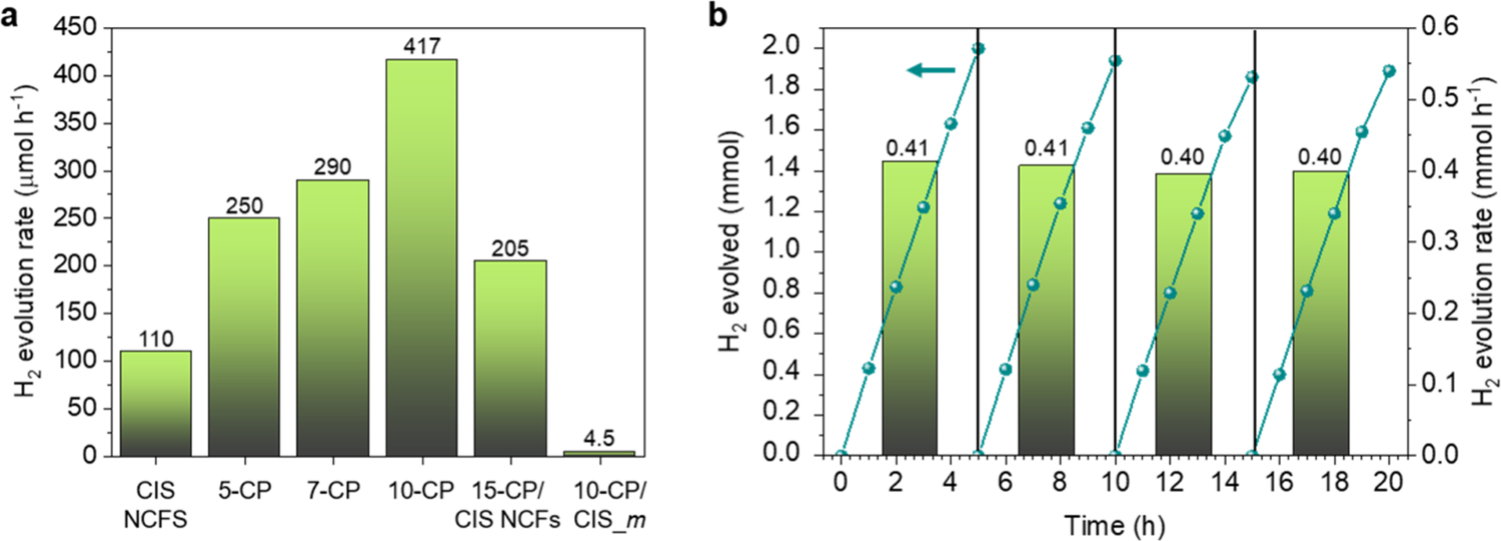
(a) Comparison between hydrogen evolution rates attained over mesoporous CP/CIS NCFs with different ratios of Co_2_P/CdIn_2_S_4_. The corresponding H_2_ evolution rate of 10-CP/CIS_*m* is also shown. (b) Time courses for photocatalytic hydrogen production (lines) and hydrogen evolution rates (columns) during the stability study of 10-CP/CIS NCFs. The H_2_ evolution rates were averaged over 5 h of irradiation.

The photocatalytic H_2_ evolution performance of the mesoporous CIS decorated with different amounts of Co_2_P was investigated in an airtight reactor cell under visible light irradiation (λ ≥ 420 nm), using triethanolamine (TEOA, 10% v/v) as the hole scavenger. Figure 4a presents a comparison of the hydrogen evolution activities of mesoporous CIS and CP/CIS NCFs. For comparison, the photocatalytic H_2_-production activities of CIS microparticle and 10-CP/CIS_*m* bulk catalysts were also evaluated under identical conditions. Strikingly, the CIS NCFs showed an H_2_ evolution rate of 110 μmol h^–1^, representing a 220-fold increase compared to that of bulk analogues (∼0.5 μmol h^–1^), thus demonstrating an enormous improvement of the H_2_ production activity. The evolution rate of H_2_ was significantly raised to 417 μmol h^–1^ by incorporating a small amount of Co_2_P (10 wt %); this activity is an enchantment by a factor of 3.8× compared to the pristine CIS NCFs. Note that, even slight decoration of Co_2_P (5 wt %) has a significant impact on the photocatalytic activity, yielding a generation rate of H_2_ ∼ 250 μmol h^–1^. Further increasing the Co_2_P concentration (15 wt %) causes a slight decline in photocatalytic performance (205 μmol h^–1^), presumably due to the light-shielding effect of Co_2_P (incident light absorbed by Co_2_P and not by CIS) and rapid electron–hole recombination at the interface of catalyst. Evidently, the hybridization between Co_2_P and CIS improves the charge separation efficiency and increases the availability of surface-reaching electrons for water reduction, as confirmed by photoluminescence and electrochemical measurements (see below). In striking contrast to mesoporous 10-CP/CIS NCFs, the 10-CP/CIS_*m* bulk catalyst with identical composition manifests aggravated reactivity toward H_2_ evolution with a respective rate of only 4.5 μmol h^–1^. All of these results consistently show that the formation of Co_2_P–CIS heterostructures with large internal surface area and nanograin composition increases the number of active sites and shortens the diffusion path of photogenerated carriers to the solid/liquid interface, thus boosting intrinsic photocatalytic performance. Moreover, the organic molecules remaining in pores (ca. 9 wt %) have a negligible effect on the accessibility of the pore surface and, thus, on the active sites of catalyst. As a comparison sample, Co_2_P particles alone showed no photocatalytic hydrogen evolution activity under identical conditions (results are not shown). Also, control experiments indicated that no hydrogen was detected in the absence of a catalyst or in the dark, verifying that the catalyst and light irradiation were indispensable for the photocatalytic reduction of water. To identify the origin of hydrogen evolution, we intentionally performed a controlled photocatalytic reaction over 10-CP/CIS NCFs in the presence of 0.1 M sodium iodate (NaIO_3_) and 10% (v/v) TEOA, which behaved as electron and hole scavengers, respectively. Since the reduction of NaIO_3_ is a thermodynamically more viable reaction than the reduction of water (the reduction potential is 1.2 V for NaIO_3_ versus 0 V for water, at pH 0), studying photocatalysis for iodate reduction enables investigation of the competitive TEOA oxidation effect on the hydrogen formation. It has been suggested that TEOA oxidation may produce acetaldehyde (CH_3_CHO) and diethanolamine (HN(CH_2_CH_2_OH)_2_), where sequential oxidation of these products could generate hydrogen.^39,40^ Interestingly, through this experiment no hydrogen evolution was detected (by gas chromatography) even after a 3 h illumination period, confirming conclusively that the hydrogen product exactly generated by water reduction reaction.

We also determined the influence of different hole scavengers and mass loadings of the catalyst on the photocatalytic H_2_ evolution performance. The photocatalytic reactions with various sacrificial agents (keeping the catalyst load constant), such as phenol (0.35 M), KI (0.05 M), methanol (10% v/v), 5 M NaOH/ethanol (10% v/v), triethylamine (10% v/v), and triethanolamine (10% v/v), have revealed notable variations in the H_2_ evolution rates (Figure S8, Supporting Information), signifying the oxidation process as the rate-limiting step in the overall photocatalytic reaction. It was found that the photocatalytic reaction proceeds at a faster rate with TEOA as a hole scavenger. In addition, we tested the photocatalytic performance of 10-CP/CIS NCFs over different concentrations of catalyst (Figure S9, Supporting Information). A substantial increase of H_2_ generation rate was observed with increasing catalyst load, until a maximum rate was reached at 1 mg mL^–1^. Further increase of the catalyst concentration appears to be detrimental to photocatalytic efficiency due to the increased light scattering effect by the catalyst’s particles. Overall, the optimized 10-CP/CIS NCFs catalyst effectively suppresses carrier recombination, manifesting a remarkable photocatalytic H_2_ evolution rate of 20.9 mmol g_cat_^–1^ h^–1^ with an apparent quantum efficiency (AQE) of 56.1% under 420 ± 10 nm monochromatic light excitation, assuming 100% absorption of the incident photons. To the best of our knowledge, the activity of 10-CP/CIS NCFs also compares favorably with the highest reported activities for other high-performance thiospinel-based systems. Supporting Table S2 provides a comparison of the hydrogen evolution activity of our catalysts with previously reported catalysts.

The photochemical stability of the 10-CP/CIS NCFs catalyst during recycling reactions was examined, as well. After each photocatalytic test, the catalyst was retrieved through centrifugation and then redispersed in a newly prepared TEOA solution. As shown in Figure 4b, 10-CP/CIS NCFs show no perceptible decay of activity after four 5 h cycles of reuse, manifesting a constant H_2_ evolution rate of ∼406 μmol h^–1^ within experimental error (3%), thus indicating high durability and recyclability. A slight discrepancy in the photoactivity after two cycled tests may be attributed to minor photocorrosion and/or mass loss during the isolation-reuse process. Furthermore, the structural stability of the 10-CP/CIS NCFs after catalysis was further studied with various analytic techniques. Elemental EDS and XRD investigations showed insignificant variations in the chemical composition and crystallinity of the reused catalyst. EDS analysis indicated a 9.87 wt % Co_2_P content, which matches well with the composition of the fresh catalyst (ca. 9.84 wt % Ni_2_P) within experimental error (Figure S10, Supporting Information). Moreover, XPS measurements further confirmed the Co_2_P-CdIn_2_S_4_ chemical nature of the catalyst after the cycled photocatalytic tests (Figure S11, Supporting Information). It is noted that some Co suboxide (CoO_*x*_) and phosphate-like (PO_*x*_) species appear on the surface of the catalyst, possibly formed during the cycling experiments and/or sample exposure to air. Previous studies have signified the important role that surface CoO(OH) and CoPO_*x*_ layers play in the water-splitting process.^41,42^ It has been suggested that such oxide complexes may promote the dissociative adsorption of water (through the nucleophilic attack of OH^–^ on Co–O/OH) and facilitate the electrochemical hydrogen evolution reaction (by donating electrons to adsorbed H), thereby bringing more active sites to the catalyst. Nevertheless, a minor reduction in porosity after consecutive reactions was inferred from N_2_ physisorption data (Figure S12, Supporting Information). The reused catalyst possesses a slightly lower surface area (ca. 59 m^2^ g^–1^) and narrower pore sizes (ca. 3.8 nm) compared to the fresh one (ca. 75 m^2^ g^–1^ surface area; ca. 4 nm pore size). Since the photoactivity was obtained by using CIS mesostructures decorated with Co_2_P-reduction cocatalyst, some minor photocorrosion of the sulfide lattice and/or rearrangement of CIS nanoparticles in the porous structure is a viable explanation. Ultimately, a further stable long-term performance and structural durability of the catalyst are anticipated, for instance, by employing suitable oxidation cocatalysts as a complementary strategy of constructing heterostructures.

### 2.3 Charge Transfer and Reaction Mechanism

**Figure 5:**
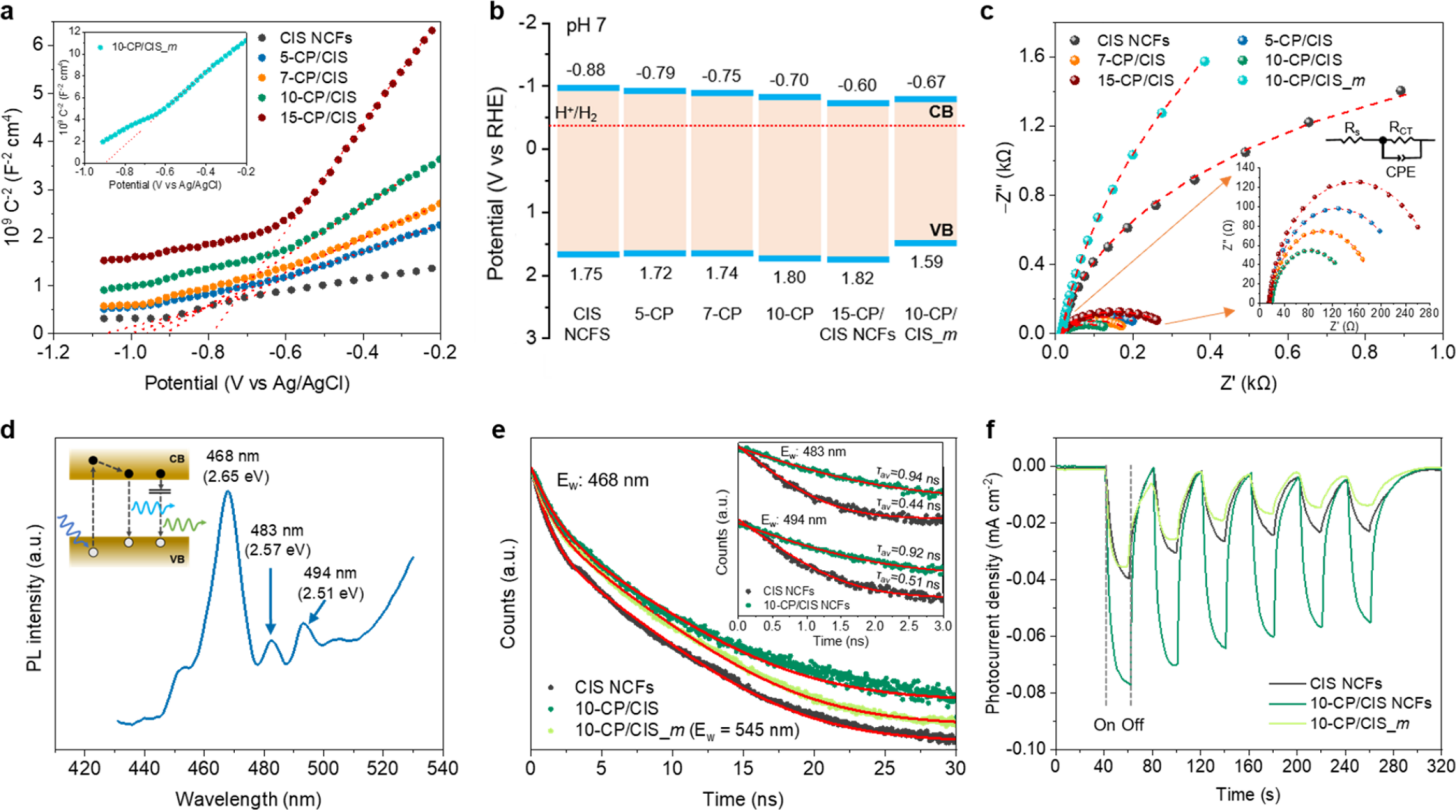
(a) Mott–Schottky plots and (b) energy band diagrams (VB: valence band, CB: conduction band, red line: H^+^/H_2_ redox potential) and (c) Nyquist plots (inset: magnification of the Nyquist plots and equivalent circuit model used to fit the EIS data) for the mesoporous CIS and CP/CIS NCFs and 10-CP/CIS_*m* samples. (d) Room-temperature steady-state PL spectrum of mesoporous CIS NCFs (excitation 380 nm). The inset shows the electron–hole recombination pathways in the CIS. (e) TRPL spectra of CIS and 10-CP/CIS NCFs at 468 nm and 10-CP/CIS_*m* at 545 nm emission wavelength using 375 nm laser pulse excitation. The inset shows the TRPL spectra of CIS and 10-CP/CIS NCFs conducted at emission wavelengths of 483 and 494 nm. The red lines are fit to the experimental data. (f) Transient photocurrent response of mesoporous CIS and 10-CP/CIS NCFs and bulk 10-CP/CIS_*m* measured at a constant potential of −1 V (vs Ag/AgCl) under visible light irradiation (λ ≥ 420 nm).

The electronic band structure of the resultant catalysts drop-cast as thin films onto fluorine-doped tin oxide (FTO) substrates was assessed through Mott–Schottky plots, that is, the reciprocal square capacitance (1/*C*_sc_^2^) vs applied potential (*E*). The flat-band potential (*E*_FB_) was obtained by measuring the *x*-axis intercept of the 1/*C*_sc_^2^ – *E* lines, as displayed in Figure 5a. All of the reported values are referred to the reversible hydrogen electrode (RHE) at pH 7 (Table 2). Apparently, all of the acquired Mott–Schottky plots show positive slopes, confirming CIS to be a n-type semiconductor, in accordance with previous studies.^43,44^ Taking into consideration that in heavily n-type doped semiconductors, the energy level of *E*_FB_ is located slightly more positive (0.1–0.3 eV) from the conduction band (CB) minimum, a rational band-edge diagram for each catalyst can be derived. The valence band energy (*E*_V_) of the catalysts was calculated by subtracting the energy band gap (as obtained from UV–vis/NIR spectra) from the *E*_FB_. The results show that the *E*_FB_ level of CIS NCFs is −0.88 V, well above the reduction potential for hydrogen (−0.41 V vs RHE at pH 7), whereas the addition of Co_2_P on the CIS surface results in remarkable changes in the band edge positions of the Co_2_P/CdIn_2_S_4_ heterostructures (Figure 5b). Specifically, Mott–Schottky plots indicated a downward trend of the *E*_FB_ level, from −0.79 to −0.60 V, with increasing Co_2_P content level (from 5 to 15 wt %). The anodic shift of *E*_FB_ is a consequence of the formation of Mott–Schottky junctions between the Co_2_P and CIS nanoparticles upon contact, leading to electron flow from CIS to Co_2_P until their Fermi levels align. The work function of Co_2_P is ∼4.6 eV vs vacuum level,^35,45^ which is much lower than the Fermi level (*E*_F_) of CIS NCFs (ca. 4.1 eV). Overall, the CIS-to-Co_2_P electron injection can generate a potential drop at the Co_2_P/CdIn_2_S_4_ interfaces (pointing from CIS to Co_2_P), resulting in a perturbation (decrease) of the donor concentration (*N*_D_) within the CIS. Such charge redistribution between CIS and Co_2_P would raise the Fermi level of Co_2_P and thus enhance the driving force for hydrogen evolution; the higher Fermi level means electrons will more easily escape from the catalyst surface to participate in redox reactions. In line with this conclusion, the inferred *N*_D_ values obtained from Mott–Schottky plots show the trend: ∼1.1 × 10^18^ cm^–3^ for CIS NCFs and ∼1.8 to 9.1 × 10^17^ cm^–3^ for CP/CIS NCFs (Table 2). The gradual concentration reduction of *N*_D_ induced by Co_2_P deposition affirms an electron delocalization from the mesoporous CIS to metallic Co_2_P, which is in agreement with the observed upshift of the CIS’s *E*_FB_ level. Compared with the mesostructured 10-CP/CIS NCFs, the less anodic shift of *E*_FB_ (−0.67 V) and lower *N*_D_ concentration loss (∼1.3 × 10^17^ cm^–3^) for the bulk 10-CP/CIS_*m* relative to its pristine CIS counterpart (*E*_FB_ ∼ −0.77 V, *N*_D_ ∼ 6.6 × 10^17^ cm^–3^, see Figure S13, Supporting Information) suggest that the electronic interactions between Co_2_P and CIS microparticles are weak, demonstrating depressed charge transfer from CIS to Co_2_P.

**Table 2:** Electrochemical Properties Derived through EIS Analysis for the Mesoporous CIS and CP/CIS NCFs, and CIS and 10 wt % Co_2_P-Loaded CIS Microparticles

	*E*_FB_			
sample	(V vs RHE, pH 7)	*E*_V_^a^	donor density (*N*_D_, cm^–3^)	*R*_ct_ (Ω)
CIS NCFs	–0.88	1.75	1.1 × 10^18^	3464
5-CP/CIS NCFs	–0.79	1.72	9.1 × 10^17^	226
7-CP/CIS NCFs	–0.75	1.74	6.5 × 10^17^	174
10-CP/CIS NCFs	–0.70	1.80	4.3 × 10^17^	129
15-CP/CIS NCFs	–0.60	1.82	1.8 × 10^17^	282
10-CP/CIS_*m*	–0.67	1.59	1.3 × 10^17^	10 925
CIS bulk	–0.77	1.58	6.6 × 10^17^	

a The valence band energy (*E*_V_) is estimated from *E*_FB_ – *E*_g_.

Electrochemical impedance spectroscopy (EIS) measurements were also conducted to determine the charge-transfer kinetics at the interface of catalysts. Figure 5c displays EIS Nyquist plots of different catalysts (drop-cast onto the FTO substrate) measured in 0.5 M Na_2_SO_4_ aqueous solution. The EIS spectra were properly fitted to a simple equivalent circuit model composed of the electrolyte resistance (*R*_s_), constant phase element (CPE), and charge-transfer resistance (*R*_ct_) (Figure 5c, inset). Obviously, all of the mesoporous Co_2_P-modified samples show much smaller *R*_ct_ values (129–282 Ω) than the pristine CIS NCFs (3464 Ω) (Table S3, Supporting Information). This suggests that coupling of CIS mesostructures with conductive Co_2_P nanoparticles led to a pronounced improvement in interfacial charge-transfer kinetics, which is favorable for promoting photocatalytic hydrogen evolution. Specifically, Co_2_P acts as an effective electronic mediator to facilitate the transfer of photogenerated electrons from CIS to the active sites. The ultrahigh conductivity of Co_2_P is elucidated by the EIS and voltage–current (*J*–*V*) investigations. EIS analysis confirmed the *R*_ct_ of the as-synthesized Co_2_P to be as low as 33 Ω (Figure S14, Supporting Information), indicating high electronic conductivity, which is essential for improved catalytic performance. In line with this, four-probe *J*–*V* measurements performed on a pressed pellet of Co_2_P reveal a linear response in the current range of −100 to +100 mA (Figure S15, Supporting Information), further supporting ohmic conduction.^46^ By comparison with the other prepared materials in this study, a considerably lower *R*_ct_ value of 129 Ω was observed for the optimized 10-CP/CIS NCFs, which implies superior interfacial charge transfer in this catalyst, consistent with its higher photocatalytic activity. Interestingly, the 10-CP/CIS_*m* bulk catalyst suffers from enormous charge transfer inefficiency, as evidenced by its high *R*_ct_ value of 10 925 Ω (Figure 5c). This accounts for poor electronic contact between Co_2_P and CIS microparticles, a behavior less relevant to the mesoporous CP/CIS materials due to the existing nanoscale junctions. Apparently, all of these results underline the importance of the synthetic process in modulating the electronic interactions and interfacial charge-transfer kinetics of Co_2_P/CdIn_2_S_4_ heterostructural catalysts.

The dynamics of photoinduced charge carrier recombination and migration were also investigated by steady-state photoluminescence (PL) and time-resolved PL (TRPL) spectroscopy to understand the intrinsic photocatalytic activity of the prepared catalysts. Figure 5d displays the PL spectrum of CIS NCFs, where the intense peak at 468 nm (2.65 eV) corresponds to the band-edge emission and the weak features at 483 nm (2.57 eV) and 494 nm (2.51 eV) are assigned as radiative relaxations of excitons through sub-bandgap trap states. Figure 5e compares the TRPL spectra recorded for the near-band-edge emission (*E*_w_) of mesoporous CIS and CP/CIS NCFs (468 nm) and bulk 10-CP/CIS_*m* (545 nm). Assuming negligible Auger-type recombination, the lifetime of charge carriers could be calculated by fitting the PL decay data to a biexponential expression: *I*(*t*) = α_1_·e^–(*t*/τ1)^ + α_2_·e^–(*t*/τ2)^, where the parameters α_1_ and α_2_ are the relative amplitudes of each lifetime component, and τ_1_ and τ_2_ are the radiative recombination lifetimes of the trap-assisted (fast) and band-to-band (slow) decay components, respectively. Through TRPL results, the average carrier lifetime (τ_av_) of 10-CP/CIS NCFs was found to be 4.06 ns, which is significantly longer than that of CIS NCFs (2.89 ns). It is obvious that electron–hole recombination is remarkably inhibited with the assistance of Co_2_P arising from a charge transfer process that entails an efficient electron migration from the bulk region to the surface. Using the average PL decay times of the samples, we further determined the rate constant of Co_2_P/CdIn_2_S_4_ interfacial electron transfer as *k*_et_ = 1/τ_10-CP/CIS_ – 1/τ_CIS_, where 1/τ_CIS_ corresponds to the exciton recombination rate (*k*_r_) and 1/τ_10-CP/CIS_ is attributed to the sum of the rate of recombination and electron transfer processes.^47,48^ Based on the above analysis, the band-edge *k*_et_ (electron transfer pathway 1, ET1) was calculated to be ∼0.1 × 10^9^ s^–1^. Moreover, the corresponding electron transfer efficiency (*n*_et_) from CIS to Co_2_P, which was acquired as *n*_et_ = *k*_et_/(*k*_et_ + *k*_r_), was found to be 29%. Note that the PL decay kinetics of the band-edge bleaching of bulk 10-CP/CIS_*m* cognate (3.57 ns) discloses a remarkably shorter average lifetime than mesoporous 10-CP/CIS NCFs, indicating that the nanoporosity and the Co_2_P/CdIn_2_S_4_ nanoscale junctions fabricated in this study are very effective for suppressing bulk carrier recombination. Another interesting observation is that the mesoporous 10-CP/CIS NCFs have a much smaller τ_1_-component of PL decay (32.8%) compared to those of unmodified CIS NCFs (79.0%) and bulk 10-CP/CIS_*m* (70.0%), which can be ascribed to its lower defect trapping. The lifetime components and the corresponding weights are summarized in Supporting Table S4. To obtain insights into the interband photoemission processes, we performed PL decay measurements at the emission channels associated with the trapping states. The trap-state PL lifetimes measured at *E*_w_ = 483 (ET2) and 494 (ET3) nm for 10-CP/CIS NCFs were 0.94 and 0.92 ns, respectively (Figure 5e, inset), which are about two times longer than those obtained for CIS NCFs (0.44 and 0.51 ns). The CIS trap-mediated electron transfer rate (*k*_et_) and efficiency (*n*_et_) into Co_2_P were calculated to be ∼1.21 × 10^9^ s^–1^ and 53% for ET2 and ∼0.87 × 10^9^ s^–1^ and 45% for ET3, respectively, which are markedly higher than these achieved by direct CB edge electron injection. The acceleration of both band-edge and trap-state PL decay kinetics demonstrates that passivation of CIS surface by Co_2_P generates specific electronic interactions between the Co_2_P nanoparticles and CIS surface-related emission centers that facilitate the separation of electron–hole pairs with ultrafast electron migration from CIS to Co_2_P. The CP/CIS NCF materials we report here undergo this phenomenon in a matter that is consistent with their efficient separation and transport of photoexcited carriers by virtue of their multipathway interfacial electron transfer processes. The same results could also be confirmed by transient photocurrent (TPC) measurements. Figure 5f shows that mesoporous 10-CP/CIS NCFs generate higher photocurrent than do CIS NCFs and bulk 10-CP/CIS_*m* for hydrogen evolution reaction. These charge-transfer dynamics have important implications for hydrogen evolution reaction because facilitate the dissociation of electron–hole pairs under light irradiation to participate in the surface redox reactions.

**Scheme 1:**
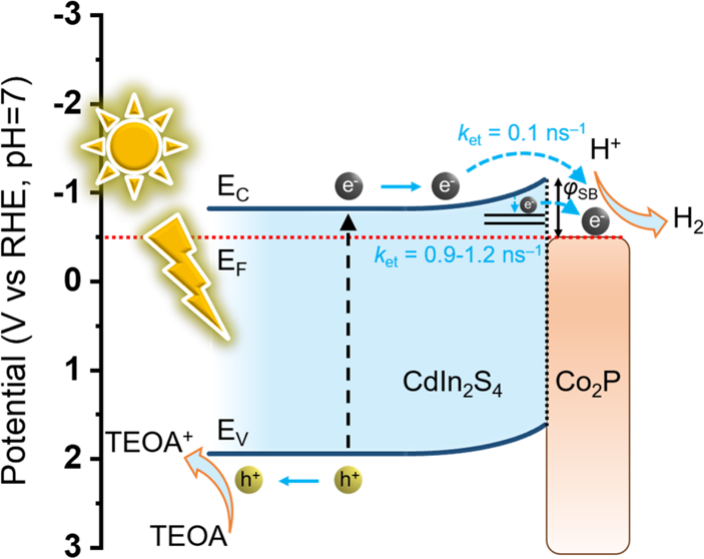
Photocatalytic H_2_ Production Mechanism and Charge-Transfer Pathways in Co_2_P/CdIn_2_S_4_ Heterostructure under Visible Light Irradiation ϕ_SB_: Schottky barrier formed at the semiconductor/metal interface, *k*_et_: interfacial electron transfer rate, *E*_V_: valence band energy, *E*_C_: conduction band energy, *E*_F_: Fermi level).

In light of the aforementioned findings, a possible charge transfer mechanism for the photocatalytic H_2_ evolution reaction over mesoporous CP/CIS NCFs is proposed in Scheme 1. Upon photoexcitation, the CB electrons of CIS tend to transfer to the Co_2_P surface. This electron transfer pathway is feasible thermodynamically due to the formation of Mott–Schottky contact between the semiconductive CIS framework and metallic Co_2_P nanoparticles, which generates a potential drop across the interface. Given the electron affinity (*x*) of the CIS NCFs (ca. 4.1 eV) and the work function (ϕ) of Co_2_P (ca. 4.6 eV), a Schottky contact (ϕ_SB_) of about 0.5 eV should be established near the interface of Co_2_P/CdIn_2_S_4_ that causes a band-edge deformation. Driven by the interfacial potential drop and high electron-withdrawing ability of metallic Co_2_P, the electrons can cross the metal/semiconductor junction and jump from the photoactivated CIS to Co_2_P, where hydrogen evolution reaction takes place, as illustrated in Scheme 1. Furthermore, surface passivation of CIS with Co_2_P can retard to a great extent the thermal relaxation of surface-reaching electrons to lower energy states (trapping states) by providing additional paths for the interfacial electron injection from trap (acceptor) states of CIS to Co_2_P nanoparticles. Here, we assumed that the trap states are located ca. 0.1–0.15 eV below the CB edge of CIS, as inferred by PL spectroscopy (Figure 5d, inset). Such unique multipathway discharging events across the Co_2_P–CdIn_2_S_4_ interface efficiently separate the photogenerated electron–hole pairs and lead to the accumulation of photoelectrons on the Co_2_P surface. During photocatalysis, the Co_2_P nanoparticles act as highly conductive electron mediators, which boost the separation and utilization of photoexcited carriers for hydrogen generation reaction. A comprehension of the CIS-to-Co_2_P electron transfer routes under light irradiation was found by ISI-XPS, TRPL, and TPC studies. Note that Co atoms in Co_2_P have been proven to be the active sites for H_2_ evolution since the Co d bands contribute to the density of states near the Fermi level and Co sites exhibit optimal H adsorption strength according to previous DFT investigations.^49^ Meanwhile, the photogenerated holes left on the VB of CIS migrate to oxidize the sacrificial agent (TEOA). Clearly, the above EIS results are commensurate with the mechanistic model illustrated in Scheme 1. Finally, we remark that because the CP/CIS NCFs are highly porous architectures (resulting from the preparation method), they have the potential to possess a high density of catalytically active sites and facilitate high mass transfer kinetics, contributing to photocatalytic hydrogen evolution improvement. Further evidence of the high permeability of 10-CP/CIS NCFs to the electrolyte was obtained from contact angle measurements. Supporting Figure S16 indicates a higher diffusion rate and better penetration of water into the mesoporous structure of 10-CP/CIS NCFs compared to the bulk 10-CP/CIS_*m* cognate; the water contact angle is almost 0° for 10-CP/CIS NCFs and ∼32° for 10-CP/CIS_*m* after diffusion for 170 μs. This verifies that the open-pore architecture is an essential feature of nanocrystal assemblies for increasing the water-catalyst interface area and thus the wettability of the catalyst. Linear sweep voltammetry (LSV) measurements on mesoporous CIS and 10-CP/CIS NCFs and bulk 10-CP/CIS_*m* catalysts under visible light (λ = 420–780 nm) illumination further assess the effect of the 3D nanostructure on the reaction kinetics. Supporting Figure S17a shows typical cathodic LSV curves obtained by using a three-electrode setup (sample-deposited working electrode, an Ag/AgCl reference electrode, and a graphite rod counter electrode) in 0.5 M Na_2_SO_4_ electrolyte. These plots show a considerably improved photocurrent density and lower onset potential for hydrogen evolution reaction over the 10-CP/CIS NCFs compared with CIS NCFs and 10-CP/CIS_*m* electrodes; for instance, at a potential of 1.4 V vs RHE (pH 7), 10-CP/CIS NCFs deliver a photocurrent density of −13.1 mA cm^–2^ vs −4.0 and −1.9 mA cm^–2^ for CIS NCFs and 10-CP/CIS_*m*, respectively. Consistent with this observation, the derived Tafel slope of the catalysts, which is strongly related to the reaction kinetics, follows the order 10-CP/CIS NCFs (192 mV dec^–1^) < CIS NCFs (237 mV dec^–1^) < 10-CP/CIS_*m* (332 mV dec^–1^) (Figure S17b, Supporting Information). As such, the lower Tafel slope value of 10-CP/CIS NCFs demonstrates better reaction kinetics for hydrogen evolution, which is consistent with their superior photocatalytic activity. Notably, because the Tafel slope values are above 120 mV dec^–1^, these studies confirmed that hydrogen evolution reaction over the CP/CIS NCFs catalysts is governed by the Volmer process (H_2_O + e^–^ + M ⇋ M–H_ads_ + OH^–^) in neutral condition, which is related to water dissociation step, followed by the desorption step of Heyrovsky reaction (M–H_ads_ + H_2_O + e^–^ ⇋ H_2_ + OH^–^ + M).

## 3 Conclusions

In summary, we demonstrate the synthesis of highly dispersed metallic Co_2_P nanoparticles on mesoporous CdIn_2_S_4_ nanocrystal assemblies to maximize their photocatalytic performance for water splitting and hydrogen production. The large accessible surface area and small grain sizes (ca. 5–7 nm diameter size) of the CdIn_2_S_4_ mesostructure along with the small size of the Co_2_P nanoparticles (ca. 6–7 nm in diameter) contribute to a close contact interaction that entails strong carrier separation and migration. A combination of spectroscopic and (photo)electrochemical characterization techniques disclose the formation of Mott–Schottky heterojunctions between the Co_2_P/CdIn_2_S_4_ interfaces that reinforce the photoexcited electron migration from both valence band and surface trap states of semiconductive CdIn_2_S_4_ to metallic Co_2_P, allowing better utilization of the charge carriers for photoredox reactions. As a result, the optimized photocatalyst containing 10 wt % Co_2_P demonstrates an excellent photocatalytic performance with a respective H_2_ evolution rate of 20.9 mmol g_cat_^–1^ h^–1^ and an AQE of 56.1% at 420 nm, which greatly surpasses those of many previously reported thiospinel-based photocatalysts. Moreover, the mesoporous framework of Co_2_P/CdIn_2_S_4_ nanocrystals demonstrated remarkable chemical and structural stability, leading to sufficient preservation of their catalytic activity in long-term operation. This work demonstrates a comprehensive understanding of the synergistic regulation of the charge transport paths in metal sulfide nanostructures toward high-efficiency photocatalytic H_2_ production. Therefore, these findings could provide a way to improve the intrinsic activity, active site density, and electrical transport properties of photocatalysts for energy conversion applications.

## 4 Materials and Methods

### 4.1 Synthesis of CdIn_2_S_4_ Nanoparticle Assemblies

The synthetic procedures for mesoporous CdIn_2_S_4_ nanoparticle assemblies (CIS NCFs) are as follows:^19^ First, 5–6 nm-sized CdIn_2_S_4_ nanoparticles were synthesized through a reflux reaction between Cd (1 mmol) and In (2 mmol) nitrates (>98% Sigma-Aldrich), 3-mercaptopropionic acid (24 mmol, 3-mpa, >99% Alfa Aesar), and thioacetamide (10 mmol, >98% Sigma-Aldrich) in 50 mL of ethylene glycol (>99% Fisher Scientific) at 150 °C for 6 h. After reaction, the CdIn_2_S_4_ nanoparticles were collected by centrifugation with addition of isopropyl alcohol, washed with copious amounts of water/ethanol (1:1 v/v) mixture, and dried at 40 °C for 24 h. The obtained thiol-capped CdIn_2_S_4_ nanoparticles were dispersed in deionized (DI) water to form a homogeneous yellow colloidal solution (100 mg mL^–1^). For the preparation of the mesoporous CdIn_2_S_4_ frameworks, 2.5 mL of a CdIn_2_S_4_ colloidal solution was suspended in 5 mL of polyoxoethylene-*b*-cetyl ether (Brij-58, Sigma-Aldrich) block copolymer aqueous solution (10% w/v) under vigorous stirring at room temperature for 1 h. Then, 1.2 mL of 3% v/v H_2_O_2_ solution was slowly added to polymerize the nanoparticles, and the resulting gel mixture was heated at 40 °C under static conditions (typically within 4–5 days) to evaporate the solvent. To remove the polymer template, the as-obtained CdIn_2_S_4_/polymer mesostructure was washed with 20 mL of warm DI water and ethanol (∼40 °C) for 2 h (three times with water and two times with ethanol). The yellow product was collected by filtration, washed several times with ethanol, and dried at 60 °C for 24 h.

For reference, bulk CdIn_2_S_4_ microparticles were also prepared by hydrothermal reaction of Cd and In nitrates and thioacetamide (1:2:10 in molar ratio) in 20 mL of water at 150 °C for 12 h. The product was centrifuged, washed several times with water and ethanol, and dried at 60 °C for 24 h.

### 4.2 Synthesis of Co_2_P Nanoparticles

Co_2_P nanoparticles were prepared using a hydrothermal reaction by modifying previously reported procedures.^35,50^ Briefly, Co(NO_3_)_2_ (0.5 mmol) and red phosphorus (5 mmol, >98% Sigma-Aldrich) were added in 20 mL of DI water and ultrasonicated for 1 h to form a homogeneous suspension. Then, the mixed solution was transferred to a 25 mL Teflon-lined autoclave and heated in an oven at 210 °C for 21 h. The obtained black precipitate was collected by centrifugation, washed several times with DI water and ethanol, and dried at 60 °C for 24 h.

### 4.3 Synthesis of Co_2_P/CdIn_2_S_4_ Heterostructures

Mesoporous Co_2_P/CdIn_2_S_4_ heterostructures (CP/CIS NCFs) were prepared by using a wet-chemical deposition technique. In a typical synthesis of 10 wt % Co_2_P-modified sample, 10 mg of Co_2_P were suspended in 60 mL of DI water/isopropanol (IPA, ≥99.8% Honeywell) mixed solution (2:1 v/v) under ultrasonication for 1 h. In a separate vial, 90 mg of mesoporous CIS NCFs were dispersed under vigorous stirring in 50 mL of DI water/IPA solution (2:1 v/v). Next, the two solutions were mixed in a 100 mL beaker, and the resulting dark green mixture was left under stirring for another 1 h at room temperature. The final product was retrieved by centrifugation, washed with ethanol, and dried at 60 °C for 24 h. For the synthesis of 5, 7, and 15 wt % Co_2_P-modified samples, the amounts of Co_2_P are 5, 7, and 15 mg and the amounts of CdIn_2_S_4_ are 95, 93, and 75 mg, respectively.

For comparison purposes, a Co_2_P/CdIn_2_S_4_ bulk reference sample (10-CP/CIS_*m*) was also prepared by depositing 10 wt % Co_2_P nanoparticles on CdIn_2_S_4_ microparticles.

### 4.4 Physicochemical Characterization

Powder XRD patterns were collected by using a PANalytical X’Pert Pro MPD X-ray diffractometer operated at 45 kV and 40 mA with Cu Kα radiation (λ = 1.5418 Å). TGA was performed on a Discovery TGA5500 system (TA Instruments) under N_2_ flow (∼200 mL min^–1^) with a heating rate of 10 °C min^–1^. The EDS spectra and FESEM images were acquired with a JEOL JSM-IT700HR scanning electron microscope operated at 20 kV. Data acquisition was performed on at least 10 different regions of every sample using a 60 s accumulation time. TEM images were obtained on a JEOL JEM-2100 electron microscope (LaB_6_ filament) operated at an accelerating voltage of 200 keV. Samples were prepared by suspending fine powders in ethanol by using sonication and then drop-casting on a carbon-coated Cu grid. XPS measurements were carried out on a SPECs spectrometer with a Phoibos 100 1D-DLD electron analyzer, using Al Kα radiation source (1486.6 eV). Prior to XPS measurements, the samples were pressed to form a pellet. All binding energies were corrected with respect to the C 1s (284.8 eV) signal of adventitious carbon. The relative atomic composition was determined from the acquired spectra after background subtraction by integrating the In 3d_5/2_ and Co 2p_3/2_ peaks and dividing them by their sensitivity factor. Peak fitting of XPS spectra was performed using the SpecsLab Prodigy software. In situ XPS experiments were conducted under dark and light irradiation conditions using a 100 W visible-light-emitting diode. UV–vis/near-IR diffuse reflectance spectra were collected with a Shimadzu UV-2600 optical spectrophotometer, using BaSO_4_ as the 100% reflectance standard. Diffuse reflectance data were converted to absorbance with the Kubelka–Munk function: α/*S* = (1 – *R*)^2^/(2*R*), where *R* is the reflectance, and α and *S* are the absorption and scattering coefficients, respectively. N_2_ physisorption measurements were performed at −196 °C on a Quantachrome NOVA 3200*e* sorption analyzer. Prior analysis, all samples were degassed at 100 °C under vacuum (<10^–5^ Torr) for 12 h. The specific surface areas were calculated applying the Brunauer–Emmett–Teller (BET) method^51^ on the adsorption data in the relative pressure (*P*/*P*_o_) range of 0.04–0.24, and the total pore volumes were calculated from the N_2_ adsorbed volumes at *P*/*P*_o_ = 0.98. The pore size distribution plots were obtained by fitting the adsorption isotherms to non-local density functional theory (NLDFT) model.^52^ Steady-state and time-resolved photoluminescence measurements were carried out on an Edinburgh FS5 spectrofluorometer.

### 4.5 Electrochemical Characterizations

The electrochemical experiments were conducted in a single-channel VersaSTAT 4 electrochemical workstation (Princeton Applied Research). The electrochemical cell consists of a working electrode, a Ag/AgCl (saturated KCl solution) reference electrode, and a Pt wire counter electrode. The working electrodes were prepared through drop-casting of the samples on fluorine-doped tin oxide (FTO, 10 Ω sq^–1^) glass substrates. Briefly, 10 mg of as-synthesized catalyst was homogeneously dispersed in 1 mL of ethanol by ultrasonication for about 1 h. Then, 100 μL of the suspension was drop-cast on an FTO substrate and dried at 60 °C for 1 h. Mott–Schottky plots were reordered in 0.5 M Na_2_SO_4_ aqueous solution (pH = 6.8) using 1 kHz with a 10 mV AC voltage amplitude. All potentials were referred to a reversible hydrogen electrode (RHE), using the following equation:
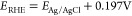
1where *E*_Ag/AgCl_ is the measured potential in the Ag/AgCl scale.

The donor concentration (*N*_D_) of the catalysts was calculated from the Mott–Schottky plots, according to the following equation:
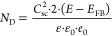
2where *C*_sc_ is the space charge capacitance, *E* is the applied potential, *E*_FB_ is the flat-band potential, ε is the dielectric permittivity of CdIn_2_S_4_ (6.6),^53^ ε_0_ is the dielectric permittivity in the vacuum (8.8542 × 10^–14^ F cm^–1^), and *e*_0_ is the elementary charge (1.602 × 10^–19^ C).

Electrochemical impedance spectroscopy (EIS) measurements were conducted in a frequency range of 1 Hz to 10 kHz with an applied voltage of −1.2 V (vs Ag/AgCl) and a modulation amplitude of 10 mV. The EIS data were fitted using ZView software. Transient photocurrent curves were recorded under chopped visible light irradiation at a fixed bias of 1 V in a 0.5 M Na_2_SO_4_ electrolyte. Polarization curves were recorded by linear sweep voltammetry (LSV) at a scan rate of 50 mV s^–1^ under visible LED light irradiation. LSV measurements were performed in a three-electrode cell in 0.5 M Na_2_SO_4_ solution with the catalyst-modified FTO substrate as a working electrode and Ag/AgCl (saturated KCl solution) and graphite rod as the reference and electrode, respectively. The electrolyte resistance (*R*_s_) was determined using EIS measurements and used for the *iR* correction of corresponding polarization curves.

### 4.6 Photocatalytic Hydrogen Production Study

The photocatalytic hydrogen production experiments were carried out in a custom-built Pyrex photoreactor sealed with silicone rubber septa. Before irradiation, 20 mg of photocatalyst was suspended in 20 mL of a water/triethanolamine mixed solution (9:1 v/v), and the suspension was purged with argon gas for at least 30 min to remove dissolved air. A xenon lamp (300 W, Variac Cermax) equipped with a UV cutoff filter (λ ≥ 420 nm) was used as a light source and a water-cooling system was employed to maintain the temperature of the suspension at 20 ± 2 °C. The photocatalytic hydrogen production activity was quantified by sampling 0.1 mL of gas and the H_2_ concertation was analyzed using a Shimadzu GC-2014 gas chromatograph (Ar carrier gas) equipped with a thermal conductivity detector (TCD).

The apparent quantum efficiency (AQE) was determined through the quantification of the evolved hydrogen at 420 ± 10 nm monochromatic light, according to the following equation:
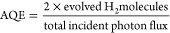
3The power density of the incident light was evaluated with a StarLite power meter, using an FL400A-BB-50 thermal detector (Ophir Optronics Ltd.).

## Supporting Information Available

EDS data, PL lifetime, electrochemical data, XRD patterns, XPS spectra, N_2_ adsorption–desorption isotherms and catalytic data of mesoporous CIS and CP/CIS NCFs, and bulk 10-CP/CIS_*m*; photocatalytic H_2_-production activity data of different thiospinel-based catalysts; TGA profiles of mesoporous CIS NCFs; XRD pattern, UV–vis spectrum, EIS Nyquist plot, and *J*–*V* plot of Co_2_P nanoparticles; FESEM images and Mott–Schottky plot of bulk CIS; and contact angle images and polarization curves of mesoporous CIS and 10-CP/CIS NCFs, and bulk 10-CP/CIS_*m* (PDF)
