# Correction to “Efficient Visible Light Photocatalytic Hydrogen Evolution by Boosting the Interfacial Electron Transfer in Mesoporous Mott–Schottky Heterojunctions of Co_2_P-Modified CdIn_2_S_4_ Nanocrystals”

**DOI:** 10.1021/acsaem.4c01594

**Published:** 2024-07-03

**Authors:** Evangelos K. Andreou, Ioannis Vamvasakis, Gerasimos S. Armatas

In our original article, the following Funding statement was added: The open access publishing of this article is financially supported by HEAL-Link.

