# Supplementary material for: Efficient Visible Light Photocatalytic Hydrogen Evolution by Boosting the Interfacial Electron Transfer in Mesoporous Mott–Schottky Heterojunctions of Co2P-Modified CdIn2S4 Nanocrystals
Source: ACS Appl Energy Mater. 2024 May 21;7(11):4891–903. doi: 10.1021/acsaem.4c00710 (PMC11192152; doi:10.1021/acsaem.4c00710)
Supplement: Supplementary file 1 — ae4c00710_si_001.pdf [file ae4c00710_si_001.pdf]

Supporting Information

Efficient Visible-Light Photocatalytic Hydrogen Evolution by Boosting the Interfacial Electron-Transfer in Mesoporous Mott-Schottky Heterojunctions of Co_2P-modified $CdIn_2S_4$ Nanocrystals

Evangelos K. Andreou[†], *Ioannis Vamvasakis*[†], *Gerasimos S. Armatas*[†,*]

[†]*Department of Materials Science and Engineering, University of Crete, Heraklion 70013, Greece*

*E-mail: garmatas@materials.uoc.gr

Supporting Tables

Table S1. Chemical composition of as-prepared Co_2P NPs and mesoporous CIS and CP/CIS NCFs.

Sample	Cd (at%)	In (at%)	S (at%)	Co (at%)	P (at%)	Co_2P loading[a)] (wt%)
Co_2P NPs	-	-	-	61.4	38.6	-
CIS NCFs	14.5	29.1	56.4	-	-	-
5-CP/CIS NCFs	15.7	30.0	47.1	5.2	2.0	4.98
7-CP/CIS NCFs	15.1	28.7	46.0	7.2	3.0	7.02
10-CP/CIS NCFs	13.9	26.4	44.6	9.6	5.5	9.84
15-CP/CIS NCFs	13.6	25.9	38.5	15.6	6.4	15.32
10-CP/CIS_*m*	13.0	25.0	48.9	9.2	3.9	10.03

[a)] Based on the EDS Co to Cd atomic ratio.

Table S2. Comparison of the photocatalytic H_2-production activities and apparent quantum efficiencies (AQEs) of different thiospinel-based photocatalysts.

Photocatalyst	Reaction Conditions	Light Source	H_2 evolution rate		AQE	Ref.
			(μmol h^{-1})	(μmol g^{-1} h^{-1})		
3 wt% Pt/ 5 wt% $Co_9S_8/CdIn_2S_4$	50 mg catalyst, 20% (v/v) TEOA	300 W Xe lamp (λ≥420 nm)	54	1084	5.5% at 420 nm	(1)
$CdIn_2S_4$@CoAl-LDH	2 mg catalyst, 10% (v/v) TEOA	300 W Xe lamp (λ≥420 nm)	1.6	793	0.2% at 450 nm	(2)
$CdIn_2S_4/In(OH)_3/Zn_2GeO_4$	50 mg catalyst, 0.35 M Na_2S, 0.25 M Na_2SO_3	300 W Xe lamp (λ≥420 nm)	71	1427	9.1% at 420 nm	(3)
1 wt% MoS_2/ $CdIn_2S4$	50 mg catalyst, 0.35 M Na_2S, 0.25 M Na_2SO_3	300 W Xe lamp (λ≥420 nm)	47	2365	5.2% at 400 nm	(4)
$CdIn_2S_4/In(OH)_3$/ NiCr-LDH	50 mg catalyst, 0.35 M Na_2S, 0.25 M Na_2SO_3	300 W Xe lamp (λ≥420 nm)	54	1093	1.7% at 420 nm	(5)
$CdIn_2S_4$/rGO/ZnS QDs	50 mg catalyst, 6% (v/v) TEOA	300 W Xe lamp (λ≥420 nm)	341	6820	19.3% at 430 nm	(6)
$CdIn_2S_4$/CNFs/ Co_4S_3	50 mg catalyst, 20% (v/v) lactic acid	300 W Xe lamp (λ≥420 nm)	1293	25870	16.3% at 420 nm	(7)
$CdIn_2S_4$/ZnS	20 mg catalyst, 0.35 M Na_2S, 0.25 M Na_2SO_3	300 W Xe lamp	75	3743	2.2% at 365 nm	(8)
$CdIn_2S_4/ZnIn_2S_4$	4 mg catalyst, 0.35 M Na_2S, 0.25 M Na_2SO_3	300 W Xe lamp (λ≥420 nm)	51	12670	18.7% at 420 nm	(9)
Co-Pi/$CdIn_2S_4$	10 mg catalyst, 20% (v/v) MeOH	500 W Xe lamp (λ≥420 nm)	73	7280	14.1% at 405 nm	(10)
$Ni_{12}P_5/CdIn_2S_4$	30 mg catalyst, 10% (v/v) TEOA	300 W Xe lamp (λ≥420 nm)	150	5010	23.5% at 400 nm	(11)
$Ni_2P/ZnIn_2S_4$	50 mg catalyst, 10% (v/v) lactic acid	300 W Xe lamp (λ>400 nm)	103	2066	7.7% at 420 nm	(12)
$CoP/ZnIn_2S_4$	20 mg catalyst, 0.35 M Na_2S, 0.25 M Na_2SO_3	300 W Xe lamp (λ≥420 nm)	175	8775	24.1% at 420 nm	(13)
$Pt/Cu_3P/ZnIn_2S_4$	50 mg catalyst, 0.35 M Na_2S, 0.25 M Na_2SO_3	300 W Xe lamp (λ≥420 nm)	128	2561	22.3% at 420 nm	(14)
$Ni_{12}P_5/ZnIn_2S_4$	50 mg catalyst, 0.35 M Na_2S, 0.25 M Na_2SO_3	300 W Xe lamp (λ≥420 nm)	113	2263	20.5% at 420 nm	(15)
CoP@$ZnIn_2S_4$	10 mg catalyst, 10% (v/v) TEOA	300 W Xe lamp (λ≥420 nm	103	10300	16.2% at 420 nm	(16)
$MoSe_2/ZnIn_2S_4$ (Z-scheme)	50 mg catalyst, 0.1 M ascorbic acid	300 W Xe lamp (λ≥420 nm	3160	63210	76.48% at 420 nm	(17)
15 wt% Ni_2P NSs/$CdIn_2S_4$	20 mg catalyst, 10% (v/v) TEOA	300 W Xe lamp (λ≥420 nm)	586	29300	61.7% at 420 nm	(18)
**10 wt%Co_2P /$CdIn_2S_4$ NCFs**	**20 mg catalyst, 10% (v/v) TEOA**	**300 W Xe lamp (λ≥420 nm)**	**417**	**20850**	**56.1 % at 420 nm**	**This work**

Table S3. Nyquist equivalent circuit fitted parameters of the prepared materials.

**Sample**	**R_s (Ω)**	**C_{dl} (F)**	**R_{ct} (Ω)**	**x^2**
CIS NCFs	15.12	37.24×10^{-6}	3464	2.2×10^{-4}
5-CP/CIS NCFs	15.3	55.37×10^{-6}	226	3.9×10^{-4}
7-CP/CIS NCFs	16.57	47.82×10^{-6}	174	1.2×10^{-4}
10-CP/CIS NCFs	18.64	54.26×10^{-6}	129	7.5×10^{-4}
15-CP/CIS NCFs	15.27	30.68×10^{-6}	282	1.5×10^{-4}
10-CP/CIS_*m*	14.68	86.82×10^{-6}	10925	2.5×10^{-4}
Co_2P nanoparticles	15.28	90.94×10^{-6}	33	7.2×10^{-4}

Table S4. Time-resolved photoluminescence decay parameters of mesoporous CIS and 10-CP/CIS NCFs, and 10 wt% Co_2P-loaded CIS microparticles (10-CP/CIS_*m*).

**Sample**	**τ_1 (ns)**	**τ_2 (ns)**	**α_1 (%)**	**α_2 (%)**	**τ_{av}[a)] (ns)**
CIS NCFs	0.74	4.28	79.0	21.0	2.89
10-CP/CIS NCFs	0.90	4.38	32.8	67.2	4.06
10-CP/CIS_*m*	1.10	4.85	70.0	30.0	3.57

[a)] The average lifetime was calculated as follows: $\tau_{av} = (\sum_i \alpha_i \tau_i^2)/(\sum_i \alpha_i \tau_i)$ (i = 1, 2).

Supporting Figures

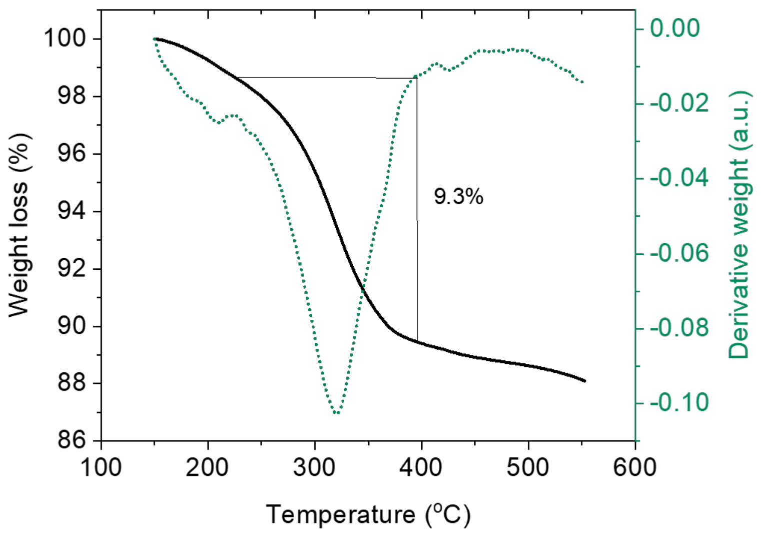

Figure S1. TGA profile (black line) and the corresponding differential thermogravimetric (DTG) curve (green line) of mesoporous CIS NCFs, showing a gradual weight loss of ~9.3% between 230 and 400 °C due to the decomposition of remaining organic residue.

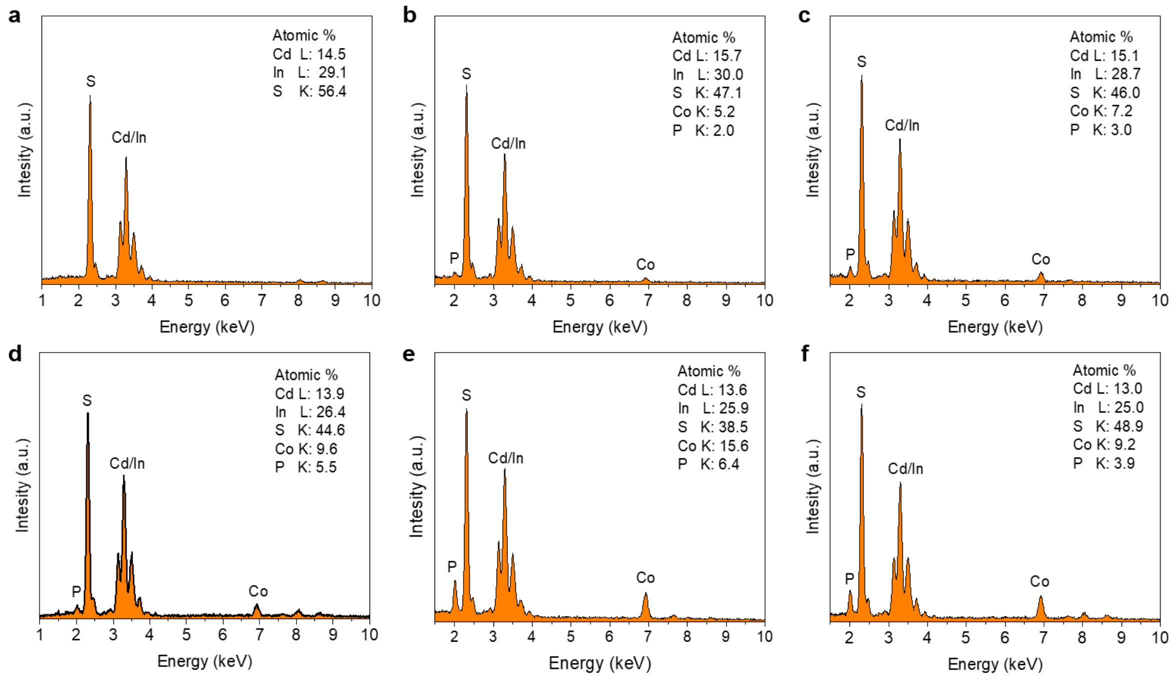

Figure S2. Typical EDS spectra of mesoporous (a) CIS and CP/CIS NCFs with (b) 5, (c) 7, (d) 10 and (e) 15 wt% Co_2P content, and (f) 10 wt% Co_2P-loaded CIS microparticles (10-CP/CIS_*m*).

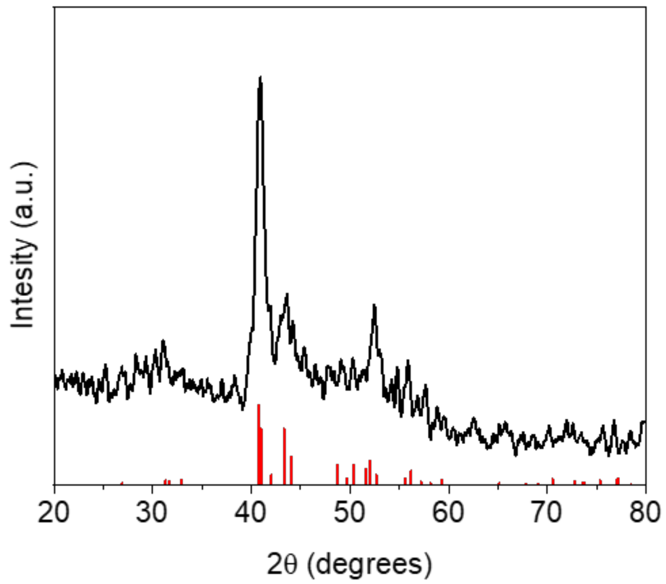

Figure S3. XRD pattern of as-made Co_2P nanoparticles. The diffraction peaks correspond to the orthorhombic structure of Co_2P (JCPDS card no. 32-0306).

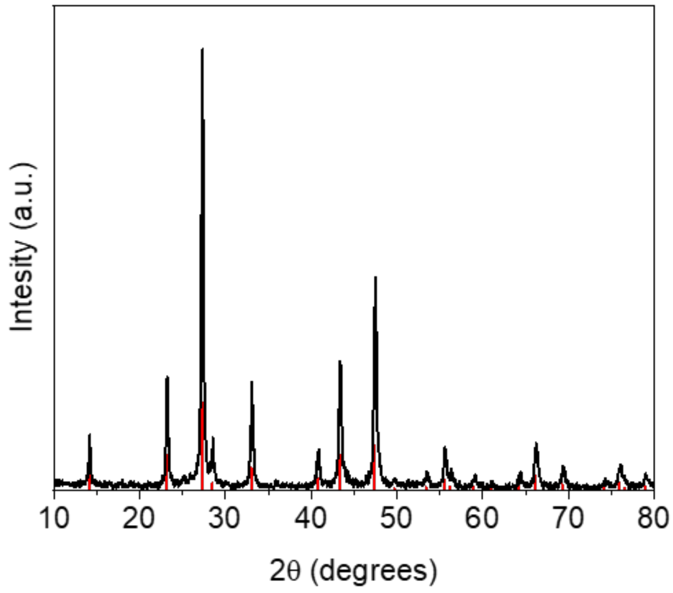

Figure S4. XRD pattern of 10 wt% Co_2P-loaded CIS microparticles (10-CP/CIS_*m*). The diffraction peaks can be assigned to the cubic structure of $CdIn_2S_4$ (JCPDS card no. 27-0060).

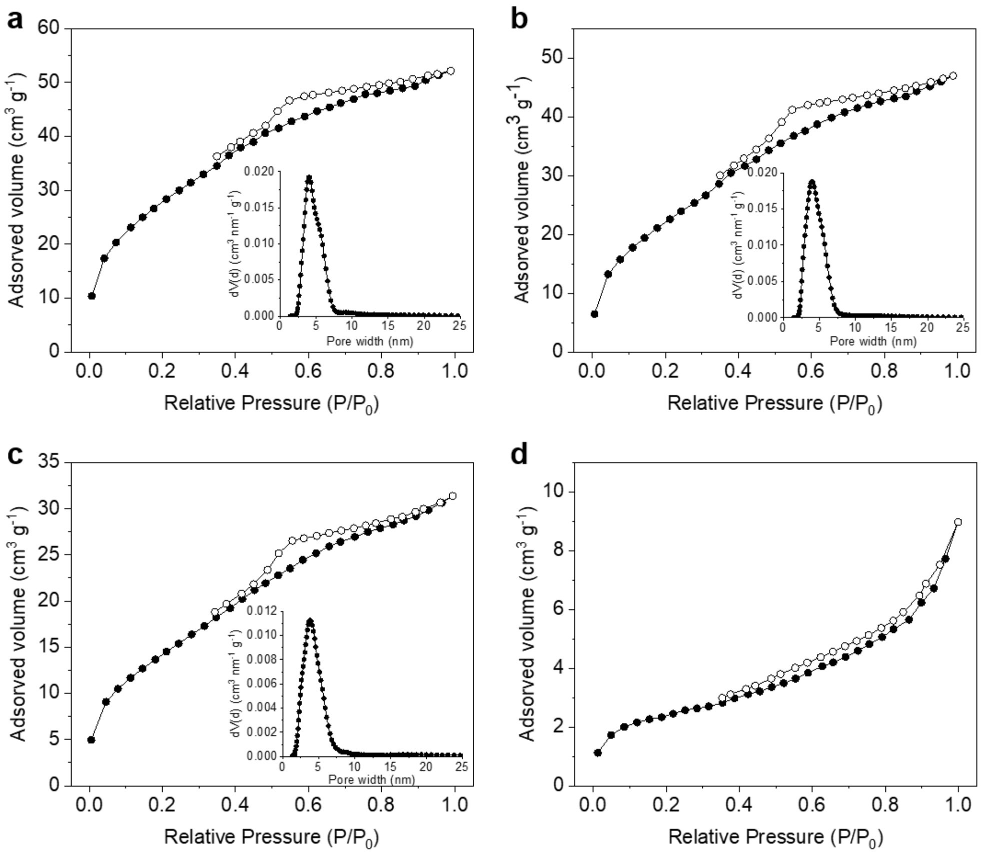

Figure S5. N_2 adsorption and desorption isotherms at −196 ºC for the mesoporous CP/CIS NCFs with (a) 5, (b) 7 and (c) 15 wt% Co_2P content, and (d) 10 wt% Co_2P-loaded CIS microparticles (10-CP/CIS_*m*). Inset: the corresponding NLDFT pore size distributions.

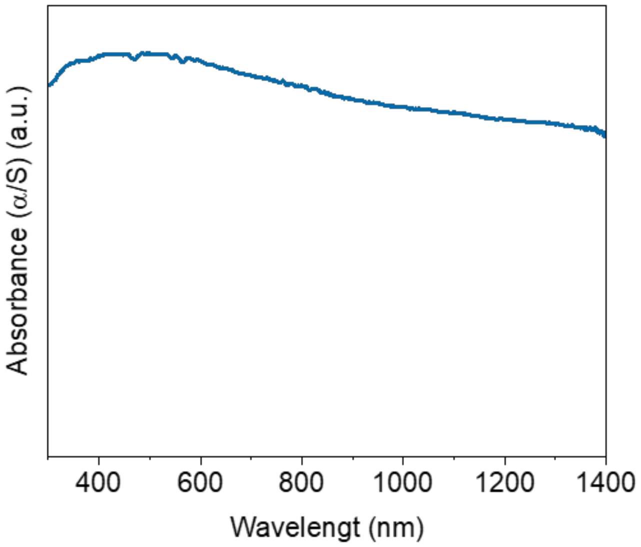

Figure S6. UV-vis/NIR diffuse reflectance spectrum of as-prepared Co_2P nanoparticles.

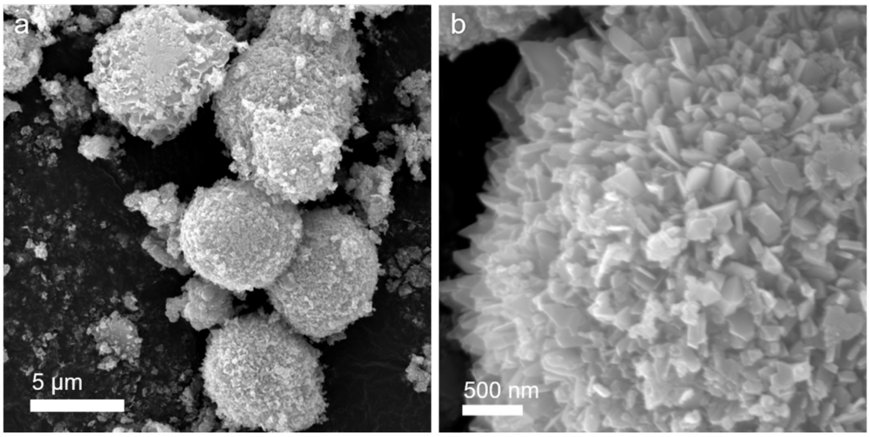

Figure S7. Representative FESEM images of bulk CIS. The images show that this sample adopts a flower-like morphology with a particle size of ~8–10 µm.

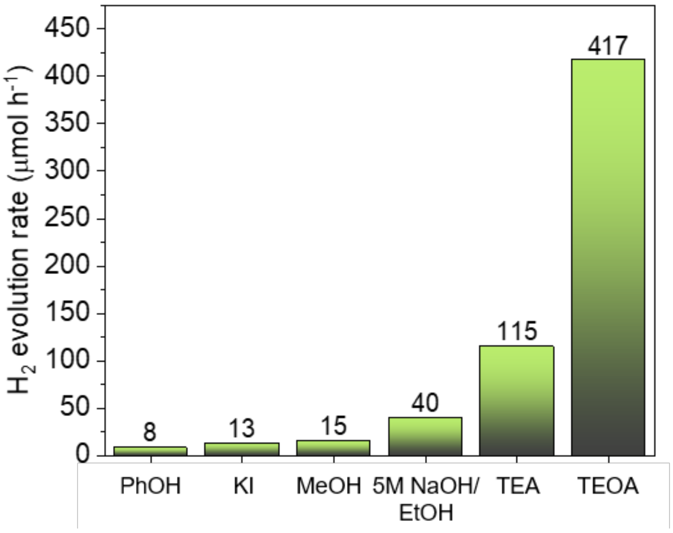

Figure S8. Photocatalytic hydrogen evolution activity for the mesoporous 10-CP/CIS NCFs using different sacrificial reagents: phenol (PhOH, 0.35 M), KI (0.05 M), methanol (MeOH, 10% v/v), 5 M NaOH/Ethanol (10% v/v), triethylamine (TEA, 10% v/v) and triethanolamine (TEOA, 10 % v/v). All the photocatalytic reactions were carried out at a fixed concentration of catalyst (1 mg mL^{-1}). Through these experiments we obtained optimal results with TEOA as the hole scavenger.

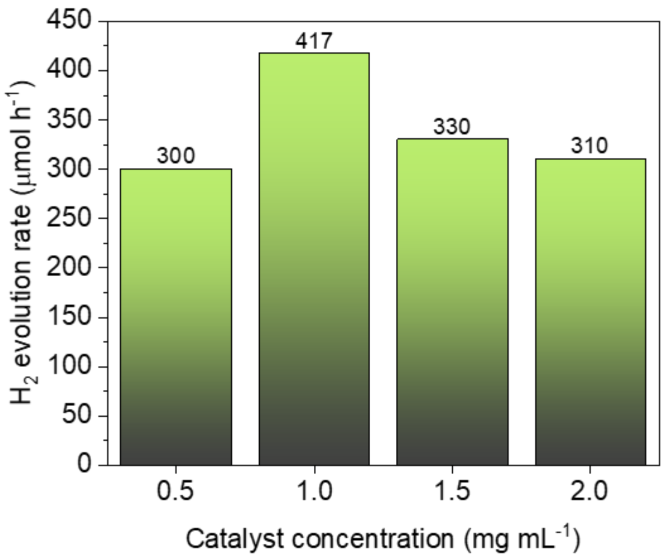

Figure S9. Photocatalytic hydrogen evolution activity for the mesoporous 10-CP/CIS NCFs using different catalyst concertation. Reaction conditions: 10–40 mg of catalyst, 20 mL aqueous solution of TEOA (10 % v/v), 300 W Xe lamp (λ ≥420 nm). Through these experiments we obtained optimal results with a 1 mg mL^{-1} catalyst dosage.

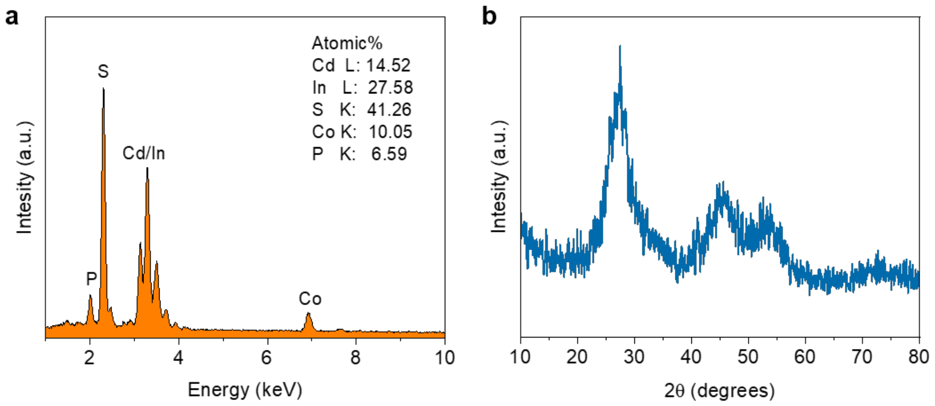

Figure S10. (a) Typical EDS spectrum and (b) XRD pattern of the 10-CP/CIS NCFs catalyst retrieved after 20 hours of photocatalytic reaction. EDS analysis indicates a 9.87 wt % Co_2P content.

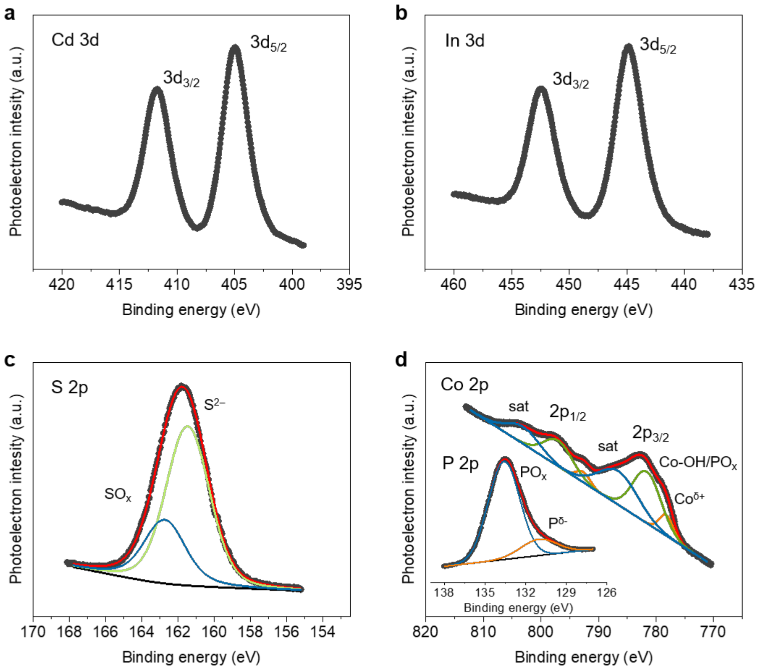

Figure S11. High-resolution XPS spectra of (a) Cd 3d, (b) In 3d, (c) S 2p and (d) Cp 2p and P 2p core-levels of the reused 10-CP/CIS NCFs catalyst. The XPS deconvoluted spectra of different chemical species are represented as blue, green and orange curves. The red lines are fits to the experimental data.

The Cd 3d XPS signal of the reused 10-CP/CIS NCFs catalyst shows a doublet peak at 404.9 and 411.7 eV binding energies due to the Cd $3d_{5/2}$ and Cd $3d_{3/2}$ core levels of Cd^{2+} ions. In the In 3d spectrum, the binding energy peaks at 444.9 and 452.5 eV are attributed to the In $3d_{5/2}$ and In $3d_{3/2}$ core-level peaks of In^{3+} oxidation state, respectively. The S 2p spectrum shows a prominent peak at 161.5 eV, corresponding to the S^{2-} valence state, and a shoulder peak at 162.4 eV, corresponding to the surface SO_x species. In the Co 2p region, the spin-orbit doublet signal at 778.2 eV (Co $2p_{3/2}$) and 792.7 eV (Co $2p_{1/2}$) is assigned to the partially charged $Co^{\delta+}$ ($0 < \delta < 2$) in Co_2P and the lines at 781.6 and 797.0 eV along with the corresponding satellite peaks at 786.5 and 803.1 eV correspond to oxidized Co species on the catalyst surface (likely as Co-OH/PO_x species). In consistence to this, the peak at 131.0 eV and the broad signal at around 133.6 eV in the P 2p region are assigned to the slightly negatively charged $P^{\delta-}$ atoms of Co_2P and to the inadequate surface oxidation (PO_x species) originating from air exposure.

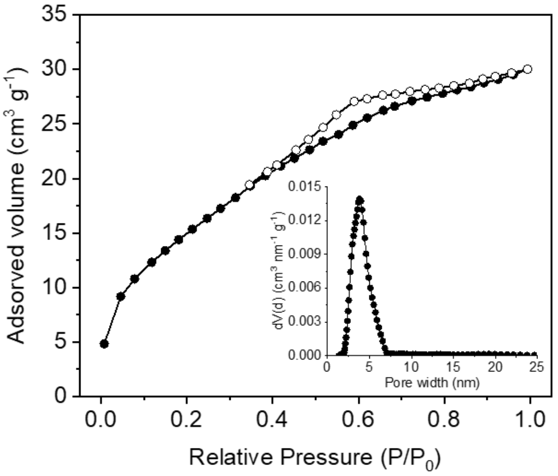

Figure S12. N_2 adsorption-desorption isotherm at –196 ºC of the 10-CP/CIS NCFs catalyst retrieved after 20 hours of reaction The inset shows the corresponding NLDFT pore size distribution. Analysis of the adsorption data gives a BET surface area of 59 m^2 g^{-1}, a total pore volume of 0.05 cm^3 g^{-1} and a NLDFT pore size of 3.8 nm.

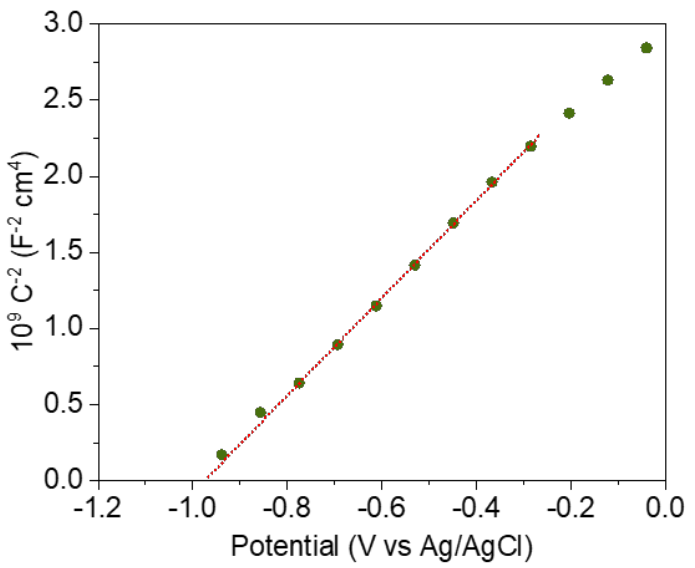

Figure S13. Mott-Schottky plot of bulk CIS. Analysis of the electrochemical data indicates a flat band potential (E_{FB}) of –0.77 V (vs RHE at pH 7) and a donor concentration (N_D) of ~6.6×10^{17} cm^{-3}.

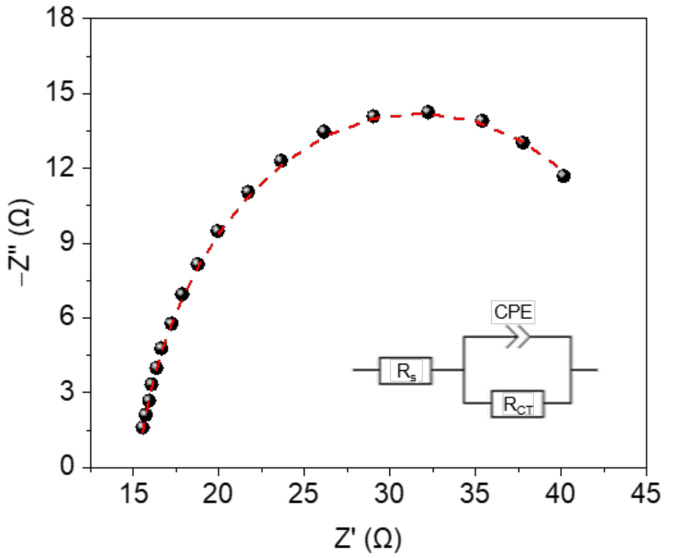

Figure S14. EIS Nyquist plot for as-prepared Co_2P nanoparticles. Inset: equivalent Randles circuit model [R_s(CPE/R_{ct})] used to fit the EIS data.

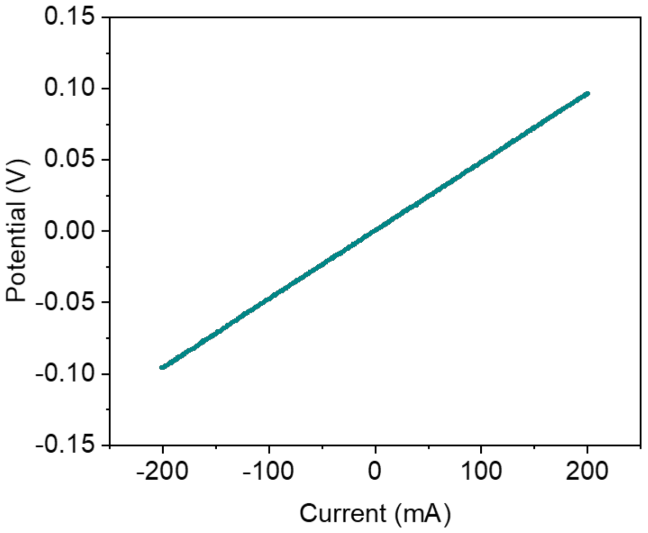

Figure S15. Voltage-current (I-V) curve of as-prepared Co_2P nanoparticles. The I-V measurement was carried out on a compressed pellet by a four-probe method.

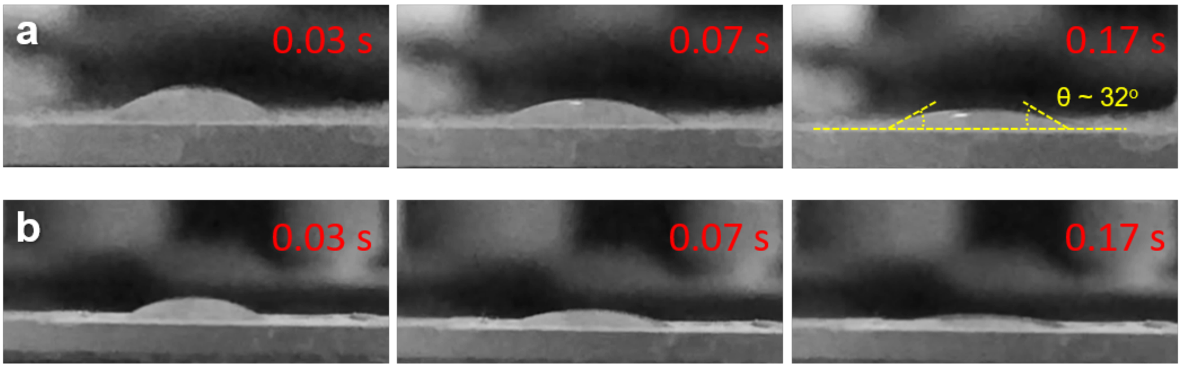

Figure S16. Contact angle measurements of the (a) bulk 10-CP/CIS_*m* and (b) mesoporous 10-CP/CIS NCFs materials. The diameter of the water droplet was approximately 2 nm.

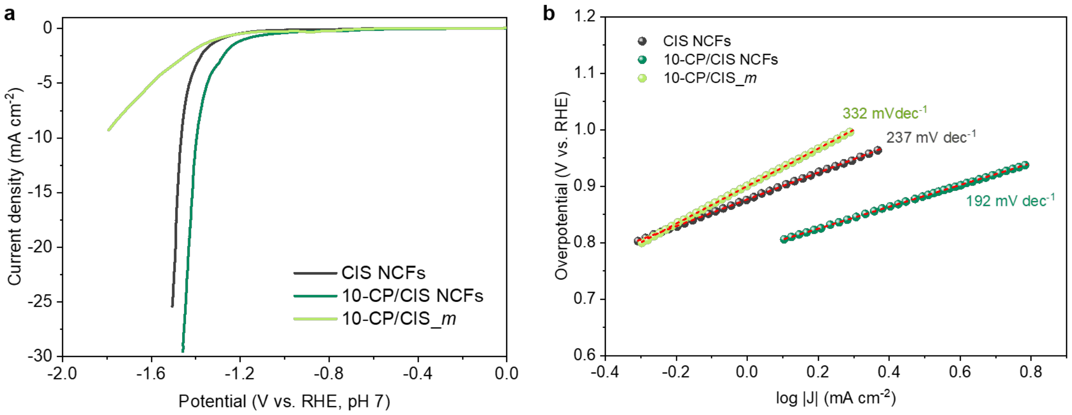

Figure S17. (a) iR-corrected polarization curves (scan rate: 50 mV s^{-1}) and (b) the corresponding Tafel plots of the mesoporous CIS and 10-CP/CIS NCFs and bulk 10-CP/CIS_*m* measured under visible light irradiation ($\lambda \geq 420$ nm). The electrochemical cell including 10-CP/CIS NCFs as the cathode displays the highest photocurrent density and onset potential for hydrogen evolution rection than those using unmodified CIS NCFs and bulk 10-CP/CIS_*m*.

References

(1) Li, C.; Zhao, Y.; Liu, X.; Huo, P.; Yan, Y.; Wang, L.; Liao, G.; Liu, C. Interface Engineering of $Co_9S_8/CdIn_2S_4$ Ohmic Junction for Efficient Photocatalytic H_2 Evolution under Visible Light. *J. Colloid. Interface Sci.*, **2021**, *600*, 794–803.

(2) Peng, L.; Yu, C.; Ma, Y.; Xie, G.; Xie, X.; Wu, Z.; Zhang, N. Self-Assembled Transition Metal Chalcogenides@CoAl-LDH 2D/2D Heterostructures with Enhanced Photoactivity for Hydrogen Evolution. *Inorg. Chem. Front.*, **2022**, *9*, 994–1005.

(3) Li, X.; Yan, X.; Zhao, N.; Zhao, J.; Lu, B.; Zhang, X.; Zhang, X.; Yu, H. Facile Synthesis of Ternary $CdIn_2S_4/In(OH)_3/Zn_2GeO_4$ Nanocomposite with Enhanced Visible-Light Photocatalytic H_2 Evolution. *J. Photochem. Photobiol. A,* **2018**, *360*, 298–305.

(4) Prabhu, Y. T.; Kumari, R.; Gautam, A.; Sreedhar, B.; Pal, U. Highly Oriented MoS_2@$CdIn_2S_4$ Nanostructures for Efficient Solar Fuel Generation. *Nano-Struct. Nano-Objects*, **2021**, *26*, 100682.

(5) Fu, R.; Gong, Y.; Li, C.; Niu, L.; Liu, X. $CdIn_2S_4/In(Oh)_3$/NiCr-LDH Multi-Interface Heterostructure Photocatalyst for Enhanced Photocatalytic H_2 Evolution and Cr(VI) Reduction. *Nanomaterials*, **2021**, *11*, 3122.

(6) Xue, C.; An, H.; Yan, X.; Li, J.; Yang, B.; Wei, J.; Yang, G. Spatial Charge Separation and Transfer in Ultrathin $CdIn_2S_4$/RGO Nanosheet Arrays Decorated by ZnS Quantum Dots for Efficient Visible-Light-Driven Hydrogen Evolution. *Nano Energy*, **2017**, *39*, 513–523.

(7) Guo, S.; Li, Y.; Xue, C.; Sun, Y.; Wu, C.; Shao, G.; Zhang, P. Controllable Construction of Hierarchically $CdIn_2S_4$/CNFs/Co_4S_3 Nanofiber Networks towards Photocatalytic Hydrogen Evolution. *Chem. Eng. J.*, **2021**, *419*, 129213.

(8) Xie, L.; Liu, G.; Suo, R.; Xie, Z.; Liu, H.; Chen, J.; Chen, J.; Lu, C.-Z. Construction of a Z-Scheme $CdIn_2S_4$/ZnS Heterojunction for the Enhanced Photocatalytic Hydrogen Evolution. *J. Alloys Compd.*, **2023**, *948*, 169692.

(9) Dang, X.; Xie, M.; Dai, F.; Guo, J.; Liu, J.; Lu, X. The in Situ Construction of $ZnIn_2S_4/CdIn_2S_4$ 2D/3D Nano Hetero-Structure for an Enhanced Visible-Light-Driven Hydrogen Production. *J. Mater. Chem. A*, **2021**, *9*, 14888–14896.

(10) Xu, J.; Li, Q.; Sui, D.; Jiang, W.; Liu, F.; Gu, X.; Zhao, Y.; Ying, P.; Mao, L.; Cai, X.; Zhang, J. In Situ Photodeposition of Cobalt Phosphate (CoH_xPO_y) on $CdIn_2S_4$ Photocatalyst for Accelerated Hole Extraction and Improved Hydrogen Evolution. *Nanomaterials*, **2023**, *13*, 420.

(11) Yang, W.; Xu, S. S.; Niu, Y.; Zhang, Y.; Xu, J. $Ni_{12}P_5$-Supported Marigold-Shaped $CdIn_2S_4$: A 2D/3D Non-Noble-Metal Catalyst for Visible-Light-Driven Hydrogen Production. *J. Phys. Chem. C*, **2023**. *127*, 4853–4861.

(12) Li, X. L.; Wang, X. J.; Zhu, J. Y.; Li, Y. P.; Zhao, J.; Li, F. T. Fabrication of Two-Dimensional $Ni_2P/ZnIn_2S_4$ Heterostructures for Enhanced Photocatalytic Hydrogen Evolution. *Chem. Eng. J.*, **2018**, *353*, 15–24.

(13) Xiang, Z.; Guan, H.; Zhang, B.; Zhao, Y. Electrostatic Self-Assembly of 2D-2D $CoP/ZnIn_2S_4$ Nanosheets for Efficient Photocatalytic Hydrogen Evolution. *J. Am. Ceram. Soc.*, **2021**, *104*, 504–513.

(14) Yang, Z.; Shao, L.; Wang, L.; Xia, X.; Liu, Y.; Cheng, S.; Yang, C.; Li, S. Boosted Photogenerated Carriers Separation in Z-Scheme $Cu_3P/ZnIn_2S_4$ Heterojunction Photocatalyst for Highly Efficient H_2 Evolution under Visible Light. *Int. J. Hydrogen Energy*, **2020**, *45*, 14334–14346.

(15) Zeng, D.; Lu, Z.; Gao, X.; Wu, B.; Ong, W. J. Hierarchical Flower-like $ZnIn_2S_4$ Anchored with Well-Dispersed $Ni_{12}P_5$ Nanoparticles for High-Quantum-Yield Photocatalytic H_2 Evolution under Visible Light. *Catal. Sci. Technol.*, **2019**, *9*, 4010–4016.

(16) Wu, B.; Liu, N.; Lu, L.; Zhang, R.; Zhang, R.; Shi, W.; Cheng, P. A MOF-Derived Hierarchical CoP@$ZnIn_2S_4$ Photocatalyst for Visible Light-Driven Hydrogen Evolution. *Chem. Commun.*, **2022**, *58*, 6622–6625.

(17) Wang, X.; Wang, X.; Huang, J.; Li, S.; Meng, A.; Li, Z. Interfacial chemical bond and internal electric field modulated Z-scheme Sv-$ZnIn_2S_4$/$MoSe_2$ photocatalyst for efficient hydrogen evolution. *Nat. Commun.,* **2021**, *12*, 4112.

(18) Andreou, E.K.; Vamvasakis, I.; Armatas, G.S. High-Performance Mesoporous Catalysts of Ultrasmall Hexagonal Thiospinel Nanocrystals for Visible-Light Hydrogen Evolution. *Adv. Mater. Interfaces,* **2024**, *11*, 2300994.
